# Bioprospecting from Marine Sediments of New Brunswick, Canada: Exploring the Relationship between Total Bacterial Diversity and Actinobacteria Diversity

**DOI:** 10.3390/md12020899

**Published:** 2014-02-13

**Authors:** Katherine Duncan, Bradley Haltli, Krista A. Gill, Russell G. Kerr

**Affiliations:** 1 Department of Biomedical Sciences, University of Prince Edward Island, 550 University Avenue, Charlottetown, PE C1A 4P3, Canada; E-Mail: kduncan@ucsd.edu; 2 Department of Chemistry, University of Prince Edward Island, 550 University Avenue, Charlottetown, PE C1A 4P3, Canada; E-Mails: bhaltli@upei.ca (B.H.); kagill@upei.ca (K.A.G.)

**Keywords:** actinomycetes, pyrosequencing, marine bacteria, *Streptomyces*

## Abstract

Actinomycetes are an important resource for the discovery of natural products with therapeutic properties. Bioprospecting for actinomycetes typically proceeds without a priori knowledge of the bacterial diversity present in sampled habitats. In this study, we endeavored to determine if overall bacterial diversity in marine sediments, as determined by 16S rDNA amplicon pyrosequencing, could be correlated with culturable actinomycete diversity, and thus serve as a powerful tool in guiding future bioprospecting efforts. Overall bacterial diversity was investigated in eight marine sediments from four sites in New Brunswick, Canada, resulting in over 44,000 high quality sequences (*x* = 5610 per sample). Analysis revealed all sites exhibited significant diversity (*H*’* =* 5.4 to 6.7). Furthermore, statistical analysis of species level bacterial communities (*D* = 0.03) indicated community composition varied according to site and was strongly influenced by sediment physiochemical composition. In contrast, cultured actinomycetes (*n* = 466, 98.3% *Streptomyces*) were ubiquitously distributed among all sites and distribution was not influenced by sediment composition, suggesting that the biogeography of culturable actinomycetes does not correlate with overall bacterial diversity in the samples examined. These actinomycetes provide a resource for future secondary metabolite discovery, as exemplified by the antimicrobial activity observed from preliminary investigation.

## 1. Introduction

Bacteria belonging to the class *Actinomycetales* are the most prolific producers of bioactive secondary metabolites in the bacterial domain. Over 10,000 bioactive compounds have been reported from actinomycetes, the largest proportion of which (~70%) are produced by members belonging to the genus *Streptomyces* [1]. More than from any other group of bacteria, actinomycete-derived secondary metabolites have had a profound impact on society, as many have been developed into effective disease treatments and account for over two-thirds of clinically relevant antibiotics [2]. Despite the success of microbial natural products in the clinic, there remains an urgent need for the discovery of new natural products to treat a variety of diseases for which there is an unmet need, such as infectious diseases caused by emerging pathogens and multidrug resistant microbes [3]. While actinomycetes have been a rich source of drug candidates over the past 60 years, reduced emphasis of natural product screening in the pharmaceutical industry and the increased difficultly in discovering novel secondary metabolites from traditional sources, such as actinomycetes from easily accessible terrestrial habitats, has led to a dearth of new natural product drug approvals [4]. Consequently, recent research efforts have focused on the exploration of underexplored habitats to discover novel bioactive secondary metabolites from the autochthonous microbiota [5,6,7,8,9]. The immensely diverse marine environment has proven to be a rich source of actinomycetes as well as novel secondary metabolites over the past decade, despite the exploration of only a small fraction of the world’s oceans [7,10,11,12,13]. Metabolic and physiological adaptation of marine microorganisms to their unique habitat exerts significant influence on bacterial speciation and consequently on the production of structurally diverse secondary metabolites; therefore, the importance of bioprospecting from novel environments cannot be underestimated [14,15].

Marine sediments are nutrient rich habitats that harbor diverse bacterial communities, which are greatly influenced by a variety of geophysical parameters [5,9,16,17,18]. These diverse bacterial communities are an untapped reservoir of genetic and metabolic diversity, and a source of novel actinomycetes, some of which have been shown to produce novel bioactive secondary metabolites [7,11,12,19,20]. The extent of actinomycete diversity in marine environments remains unclear, as does their potential to produce novel secondary metabolites. This is particularly true of sediments from temperate waters, which, have been researched to a lesser extent than those from tropical environments [14,21,22]. Recent research suggests that the actinomycete diversity of temperate sediments rivals that observed in tropical locations, and provides a less-explored resource for secondary metabolite discovery [5]. Several studies have shown exceptional actinomycete diversity from temperate waters [5,9,23,24,25]; however, these studies have focused on sediment from the Arctic Ocean, a Norwegian fjord, the NE Atlantic and Great Slave Lake, Canada. Canada boasts the longest coastline in the world, stretching for more than 243,000 km and bordering three oceans, however, actinomycete diversity present in these waters have remained largely unexplored. Therefore, the research described herein represents a study of bacterial diversity aimed at investigating the extent of actinomycete diversity in Canadian marine sediments in order to guide actinomycete culture-based bioprospecting strategies for the purpose of drug discovery. The Bay of Fundy (near Saint John, New Brunswick, Canada) was chosen as the site for this study because its intertidal areas represent an unexplored marine ecosystem which are characterized by the world’s greatest tidal variation, with maximum-recorded tides of 16.27 m [26]. 

It is well known that only a small proportion (<0.1%–1%) of bacteria present in sediments are amenable to cultivation under laboratory conditions [27]. Consequently, culture-based studies of bacterial diversity do not reveal a comprehensive picture of diversity. Molecular approaches to elucidating bacterial diversity do not rely on cultivability, thus providing a more complete picture of diversity [28,29,30,31]. Recent advances in sequencing technologies (e.g., 16S rDNA Bacterial Tag-Encoded FLX Amplicon Pyrosequencing [bTEFAP]) have enabled high-throughput sequencing of metagenomic small subunit ribosomal rRNA gene (16S rDNA) amplicons. The unprecedented sequencing depth afforded by these approaches enables a thorough examination of bacterial diversity and allows for the detection of rare phylotypes, which would be undetectable with lower throughput methods [32]. Furthermore, deep sequencing of 16S rDNA amplicons generated from metagenomic DNA isolated from marine sediments can be used to enhance our understanding of actinomycete biodiversity in marine environments by beginning to correlate this wealth of sequencing information with environmental parameters, biogeography and overall composition of bacterial communities [32,33]. The bTEFAP approach allows for unprecedented speed and economy in characterizing microbial diversity. With ever decreasing sequencing costs it is now economical to profile the bacterial diversity in habitats prior to initiating extensive isolation efforts. The concept of biodiversity translating to secondary metabolite diversity is a central tenant of natural product research, however, it is not known if overall bacterial diversity of sediments correlates with diversity of taxa with a high potential to produce bioactive secondary metabolites (e.g., actinomycetes). Determining if such a correlation exists could greatly impact bioprospecting strategies, allowing researchers to focus efforts on sediments likely to possess a high diversity of biotechnologically important taxa.

The initial aim of this study was to profile the bacterial diversity present in eight sediments collected from four locations in the Bay of Fundy, New Brunswick, using a rapid and economical culture-independent pyrosequencing-based approach. We then set out to determine the culturable actinomycete diversity present in these sediments using a combination of selective plating techniques and selective isolation media. This allowed the taxonomic diversity revealed using the two approaches to be compared in order to explore the relationship between overall microbial diversity and actinomycete diversity. Finally, to assess the biotechnological potential of actinomycetes isolated from the Bay of Fundy, we fermented selected actinomycete isolates and evaluated the corresponding fermentation extracts for the presence of metabolites with antimicrobial activity. 

## 2. Results and Discussion

### 2.1. Culture-Independent Analysis of Bacterial Diversity in New Brunswick Marine Sediments

The initial aim of this study was to characterize the bacterial diversity present in the Bay of Fundy marine sediments using a bTEFAP approach. Stringent processing of raw pyrosequencing data using mothur furnished over 44,000 high quality partial 16S rRNA gene sequences, which span entirely the V1 region of the 16S rDNA gene and extend into the V2 region. Sediment sequence libraries contained 3672–7856 sequences per sample (*x* = 5610) (Table 1). Species level (*D =* 0.03) richness (*S*), varied from 1232 to 2102 operational taxonomic units (OTUs) between sediment sample locations; when subsampled to 3500 sequences to account for variation between samples, richness varied from 976 to 1456 OTUs (Table 1). As observed richness is sensitive to sample size, the Chao1 estimator was used to estimate richness at the species level [34,35]. Richness estimates ranged from 2294 to 4419 OTUs, while subsampled data estimates ranged from 2255 to 3459 OTUs.

To calculate the degree to which the observed number of OTUs represented the predicted number of OTUs, the sampling coverage (*C*) was calculated for each sample. Sampling depth was sufficient to detect 79%–86% of predicted OTUs while in the subsampled data set 73%–82% of expected OTUs were observed (Table 1). To compare bacterial diversity between sites while taking into account evenness and richness, the Shannon Diversity Index (*H*’) was calculated, which varied from 5.41 (6B) to 6.55 (3B) (subsampled data set), with sites 4B–6B from Black Beach exhibiting lower average diversity (*H*’ = 5.54) than samples collected at the other three sites (*H*’ > 6.24). The Shannon Equitability Index (*E*), which is a measure of community evenness, showed all sites exhibited high evenness (0.76–0.90), although the bacterial communities from the Black Beach sites were slightly less even (*E* = 0.79; subsampled data) than those from the other sites (*E* > 0.88) (Table 1). 

**Table 1:** Richness and diversity estimates (*D* = 0.03) for bTEFAP 16S rRNA gene sequence libraries from New Brunswick sediment. Richness estimates are shown for data sets containing all available sequences as well as data sets subsampled to 3500 sequences.

Sample	Sample size	Average sequence length (bp)	Richness	Chao1	C (%) ^a^	Shannon diversity index (*H’*)	Shannon equitability index (*E*)
1B	6791	244	2102	3812	83	6.74	0.88
	3500	-	1412	3123	74	6.55	0.90
2B	4634	246	1555	3247	79	6.39	0.87
	3500	-	1301	2990	76	6.31	0.88
3B	5957	246	2040	4419	79	6.69	0.88
	3500	-	1456	3459	73	6.52	0.90
4B	7856	247	1841	3806	86	5.73	0.76
	3500	-	1098	2750	79	5.54	0.80
5B	4383	247	1276	2674	82	5.72	0.80
	3500	-	1108	2435	80	5.66	0.81
6B	5306	247	1264	2743	86	5.51	0.77
	3500	-	976	2255	82	5.41	0.76
7B	3672	246	1232	2294	81	6.38	0.90
	3500	-	1199	2268	80	6.36	0.90
8B	6283	244	1659	2888	86	6.38	0.86
	3500	-	1195	2403	80	6.24	0.88

^a^ Coverage = (1 − [OTU_singleton_/OTU_total_]) × 100%.

The sequencing depth obtained in this study identified high bacterial richness and resulted in sufficient sampling coverage to comprehensively describe diversity, although some bacterial diversity (18%–27%) was left undiscovered (subsampled data). Previous studies have reported richness of microbial communities in the marine environment to vary in the water column from a few hundred species per milliliter [36] to several thousand phylotypes in marine sediments [28,37]. However, comparison of observed and predicted richness between studies is challenging as the outcome is greatly influence by sequencing depth and the hypervariable region of the 16S rRNA being analyzed [38]. Therefore, comparison of diversity indices that compensate for sampling depth offer a more robust method of comparing between studies. The Shannon Diversity Index calculated for the Bay of Fundy sediments varied from 5.41 to 6.55 (subsampled data). These values are higher than those reported for 98 soil samples (~2.4–3.6) [39] and heavily polluted fresh water sediments from the Anacostia River (2.0–2.7) [40]. The diversity levels of Bay of Fundy sediments are similar to those reported from tropical freshwater, intertidal wetland and marine sediments (*H*’ 6.53–6.95) from southern China [28]. This suggests that bacterial diversity in sediments from temperate environments is approximately equivalent to the diversity in tropical sediments. From a bioprospecting perspective this suggests that temperate marine sediments can be expected to be as productive as those from the tropics. It is however, important to recognize that further sampling of temperate environments using this approach should be conducted to further support this conclusion.

**Figure 1:**
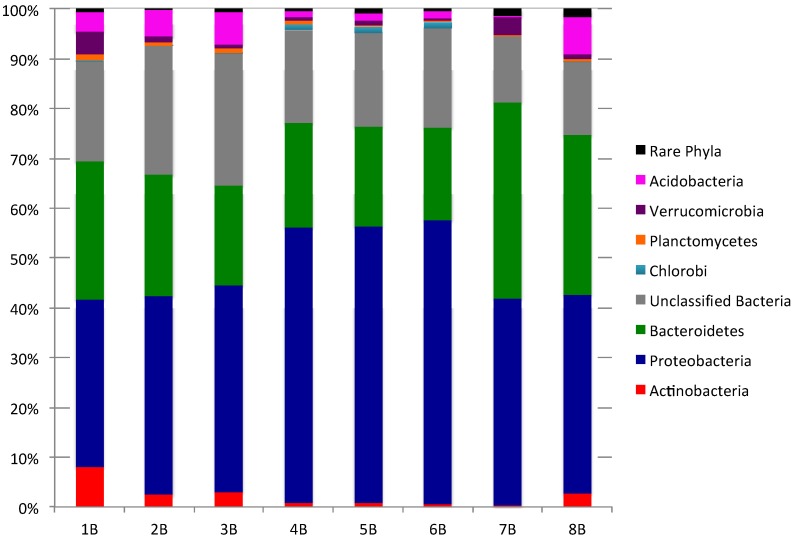
Phylum level comparison of bacterial community composition for eight sediment samples (“Rare Phyla” include all phyla comprising <1% of the total bacterial community composition).

To explore the composition of bacterial communities in each sediment sample, 16S rRNA gene sequences within each 454-sequence library were classified using mothur employing a confidence threshold of 80%. The 44,891 sequences were classified and assigned to 19 bacterial phyla with 10–15 phyla present in each sediment sample (Figure 1; Supplementary Tables S1 and S2 for “rare Phyla” that account for <1% of bacterial community). *Bacteriodetes* (18.7% to 39.5%) and *Proteobacteria* (33.6% to 56.9%) were the dominant phyla in all sediments while *Actinobacteria* (0.4% to 8.0%), *Verrucomicrobia* (0.5% to 4.5%), and *Acidobacteria* (0.1% to 7.5%), comprised a small but significant percentage of the bacterial communities. The artificial group “Rare Phyla” (<1% abundance) accounted for 0.2% to 1.5% of sequences in each sequence library. Unclassified bacteria, OTUs that could not be assigned to a phylum with an 80% confidence threshold, were prevalent in all sediments, accounting for 13.3%–26.5% of sequences.

Assessment of class level diversity identified 58 classes from all samples, with 29–44 classes identified in each sediment sample [41]. *Gammaproteobacteria* (15.3% to 49.5%) and *Flavobacteria* (12.2% to 26.8%) dominated the class level diversity in all sediments while *Betaproteobacteria* (0.5% to 10.7%), *Deltaproteobacteria* (0.2% to 8.6%), *Sphingobacteria* (0.3% to 4.5%) and *Actinobacteria* (0.4% to 8.0%) comprised a small but significant percentage of all eight bacterial communities. Bacterial communities from Chance Harbour sediments (4B–6B) could be clearly differentiated from the other sediments due to a greater prevalence of *Gammaproteobacteria* in these sediments (47.7% to 49.5% *vs.* <29.5%). Pocologan (7B) sediments could be differentiated from the other sediments based on a nearly two fold greater prevalence of *Flavobacteria* (26.9%) and *Deltaproteobacteria* (8.6%). The Mispec Bay sediment (8B) bacterial community was characterized by a higher percentage of *Betaproteobacteria* (10.7%) and *Acidobacteria*_GP4 (4.4%) compared to other sediments. Similar to 8B, Black Beach (1B–3B) also had a higher prevalence of *Acidobacteria*_GP4 compared to the other sediments (<0.4%). Similar community structure has been observed in intertidal sediments from both tropical [42] and temperate locations [43,44]. 

**Figure 2:**
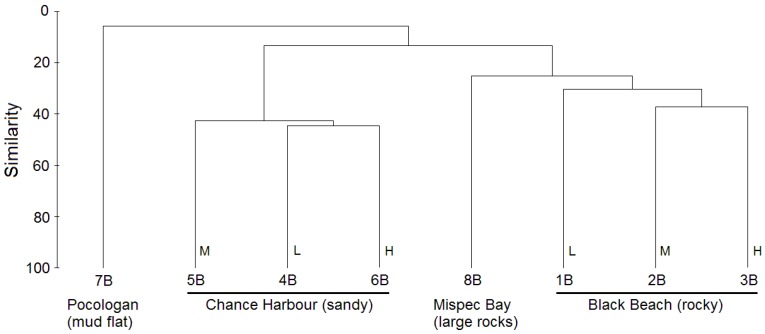
Complete-linkage cluster analysis (based on Bray-Curtis similarity matrix with square root standardization) comparing species level bacterial communities between eight sediment samples. Sediments collected at high, mid or low tidemark indicated by H, M and L, respectively.

To compare the taxonomic composition of the bacterial communities of the eight sediments, cluster analysis of species level bacterial communities was conducted. This analysis revealed that bacterial communities of the eight sediment samples clustered based on sample location (Figure 2). Sediment samples from Black Beach (1B–3B) and those from Chance Harbour (4B–6B) clustered in separate clades. Samples 1B–3B formed a clade with the Mispec Bay (8B) sample. The bacterial community of sediment 7B from Pocologan mud flat was the most distinct and did not cluster closely with any of the other samples. To determine if the four sites investigated host unique bacterial communities an analysis of similarity (ANOSIM) was conducted using PRIMER. The R statistic generated from this analysis was 1 for each of the pairwise comparisons (except for 7B and 8B which could not be compared to each other due to lack of replicates) suggesting that each site hosts a unique bacterial community; however, only the comparison between Chance Harbour and Black Beach approached statistical significance (*p* = 0.1). Collectively, the cluster and ANOSIM analyses suggest that there are differences in the bacterial communities at each site; however, further sampling would be required to confirm if trends are statistically significant.

**Figure 3:**
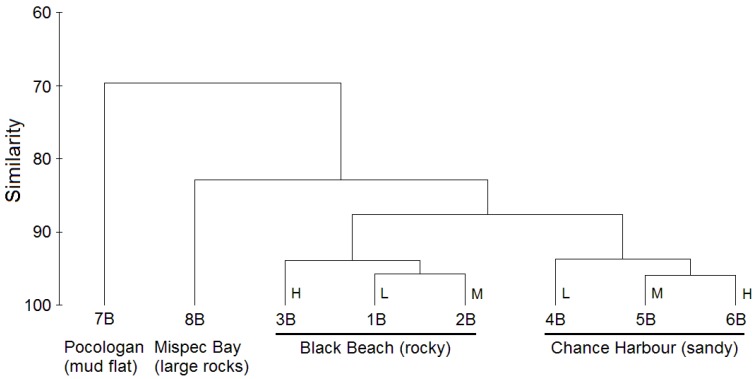
Complete-linkage cluster analysis (based on Bray-Curtis similarity matrix with square root standardization) comparing the chemical composition of eight sediment samples. Sediments collected at high, mid or low tidemark indicated by H, M and L, respectively.

To investigate the potential influence of sediment composition on the structure of sediment microbial communities a cluster analysis was performed on data obtained from sediment composition analysis (Table 2; Figure 3).

**Table 2:** Chemical analysis of sediment composition for eight sediment samples.

Sample	Concentration in parts per million (ppm)												Particle size (mm) as a percentage of total sample weight			
	S^2−^	Zn^2+^	Na^+^	Fe^2/3+^	Cu^2+^	B	Mg^2+^	Ca^2+^	K_2_O	P_2_O_2_	pH	OM (%)	<0.6	0.6–1.0	1.0–2.0	>2
1B	57	3.3	1088	233	1.7	2.3	270	248	194	51	7.2	1.6	22.2	57.5	18.9	1.4
2B	77	3	1164	236	1.7	4.1	330	314	213	45	7.9	1.3	12.4	51.8	32	3.9
3B	53	2.9	1003	220	1.7	3.3	283	259	198	39	7.7	1.4	0.5	23	72.7	3.9
4B	81	1	1095	132	0.1	1.6	192	141	111	42	7.8	0.4	4.4	34.1	61.2	0.4
5B	97	1	1264	170	0.1	1.8	214	304	122	50	7.9	0.3	2.8	20.4	75.6	1.3
6B	107	1.1	1293	147	0.2	1.9	229	163	125	46	7.7	0.3	0.7	17.1	82.3	0
7B	128	2.4	2895	575	0.3	6	508	485	387	187	7.9	1.5	14.6	5.5	79.9	0
8B	49	1.6	740	137	0.3	1.4	169	194	118	27	7.9	3.4	0.1	4.4	54	41

Interestingly, the clustering pattern in this analysis closely matches that observed in the dendrogram based on species level bacterial community composition (Figure 2). Samples again grouped by site, however, branching patterns within the Black Beach and Chance Harbour clades were slightly different than that observed in the cluster analysis based on microbial community structure. Within the Black Beach clade, the low and mid tide samples clustered together. In contrast to the clustering pattern observed in Figure 2, the low and high tide samples cluster together. The clustering pattern within the Chance Harbour clade was similar to that observed in Figure 2, with the mid tide sample clustering more closely with the high tide sample. 8B clusters with Chance Harbour samples in Figure 2 but forms an outgroup for both Chance Harbour and Black Beach sediment samples when sediment composition is compared (Figure 3). 7B was the most distinct sample in terms of sediment composition. Characteristics of 7B that contributed to its differentiation from the other samples included the highest levels among the eight sediments analyzed of phosphorous (P_2_O_5_), potassium (K_2_O), calcium, magnesium, boron, iron, sodium and sulphur (Table 2). Consistent patterns also emerged when comparing the composition of Black Beach (1B–3B) to Chance Harbour (4B–6B) sediments. Black beach sediments contained higher levels of phosphorous, magnesium, boron, copper, iron, zinc and manganese while having lower levels of sulphur compared with all three sediment samples from Chance Harbour. Furthermore, sediment from Chance Harbour had the lowest organic matter (OM) (0.3% to 0.4%) compared with all other sites which had greater than 1.3% OM. Sediment from Mispec Bay had the lowest phosphorous, iron, sodium and sulphur of all sites tested and the sediment had the largest particle size with 41% of the sediment particles being greater than 2 mm. Collectively, the overall congruence in clustering patterns observed in analyses of bacterial community composition (Figure 2) and sediment composition (Figure 3) suggest that sediment composition may influence the structure of resident bacterial communities. Canonical correspondence analysis (CCA) was preformed to statistically assess if sediment parameters influenced the composition of sediment bacterial communities (culture-independent). The community composition was based on OTUs generated from mothur analysis of pyrosequencing data. Due to the large data set, CCA was split into three groups of sediment variables to enable each variable to be tested for significance. Group one (Supplementary Figure S1: % Organic Matter (OM), pH, P_2_O_2_, K_2_O, Ca and Mg), group two (Supplementary Figure S2: B, Cu, Fe, Na, Zn, S) and group three (Supplementary Figure S3: sediment particle size: size 1 (<0.6 mm), size 2 (0.6–1 mm), size 3 (1–2 mm) and size 4 (>2 mm)). All groups of physiochemical variables were strongly correlated with the composition of bacterial communities: Group 1 F = 1.913, *p* = 0.001; Group 2 F = 1.963, *p* ≤ 0.0001; Group 3 F = 1.780, *p* = 0.016. The results of the first and second CCAs revealed similar clustering of sites as observed in Figure 2 and Figure 3. Black Beach (1B–3B) clustered with Mispec Bay (8B) and all correlated with percent OM (Figure S1), Cu and Zn (Figure S2). Chance Harbour sites (4B–6B) clustered separately, correlated with pH (Figure S1) and S (Figure S2). Pocologan (7B) sediment was quite distinct from all other sites in bacterial community composition. Sediment particle size (Group three CCA) appeared to only weakly correlate with bacterial community composition within each site.

These analyses suggest that the physiochemical composition of Bay of Fundy sediments shape the structure of species level bacterial communities. While the clustering within highly similar clades was somewhat variable, the major clustering relationships between samples were consistent between analyses conducted on species level bacterial community analysis and sediment composition. Similar correlations have been observed in studies of soils, fresh water sediments, hyper-saline soils and sediment, and marine sediments from the Arctic Ocean [18,23,45]. The results presented here further extend these observations to intertidal marine sediments. It has been suggested that the physiochemical conditions in sediments are affected by their geochemical composition and consequently influence indigenous microbial communities [46]. In contrast to physiochemical composition, location within the intertidal zone did not appear to significantly influence the structure of microbial communities. It was found that bacterial communities at each tidal location were highly similar. The lack of distinction between these communities may be a result of the local homogenizing effects of the daily tides; however, the limited sample size of this study does not allow for definitive conclusions regarding the effect of intertidal zone position on bacterial community structure.

### 2.2. Culture-Independent Analysis of Actinomycete Diversity

**Figure 4:**
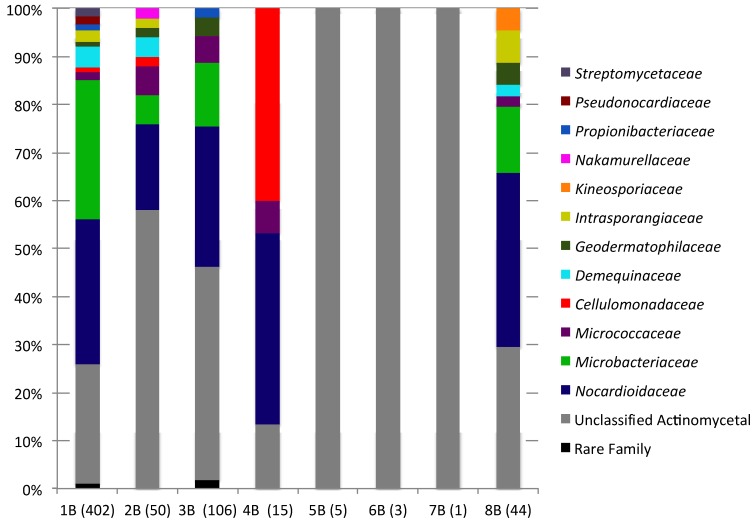
Family level bacterial community composition within the order *Actinomycetales* of eight sediment samples. Numbers in brackets indicate the total number of *Actinomycetales* sequences in each sample.

Actinomycete sequences accounted for 0.3% (7B)–5.9% (1B) of each sediment sequence library. Actinomycete sequences were rare in 4B–7B sequences libraries (1–15 sequences), while 1B–3B and 8B contained a moderate number of sequences (44–402). In total 14 actinomycete families were detected (in addition to unclassified actinomycetes) in the New Brunswick sediments and between 15 (1B) and 1 (7B) actinomycete families were detected in each sediment sample. The greatest family level diversity was observed in sediments from Black Beach (1B–3B) and Mispec Bay (8B). However, the greater diversity observed in these samples is likely due to the presence of greater number of actinomycete sequences. Family level composition of actinomycete communities is summarized in Figure 4 and Supplementary Table S3. 

*Nocardioidaceae* was the dominant actinomycete family in five of seven samples (1B–4B and 8B), accounting for 18%–40% of actinomycete communities. *Micrococcaceae* were detected in the same samples (1B–4B and 8B) and accounted for 1.7%–6.7%. In contrast to all other sediments, sample 4B exhibited a high prevalence of *Cellulomonadaceae* (40%). Unclassified actinomycetes were present in all samples (13.3%–100%), these sequences may represent novel lineages within the *Actinomycetales;* however, longer 16S rDNA sequences would be needed to confidently determine the taxonomic novelty of these OTUs.

Actinobacteria have been observed within marine environments, increasingly using next generation sequencing (due to the decreasing costs of generating large sequencing data sets) [28,47,48,49], as well as more traditional culture-independent clone library methods [24,50]. *Actinobacteria* abundance varied significantly between Bay of Fundy sediments (≤1%–9.45%) but in general is comparable to levels observed in other studies from a variety of habitats. For example, culturable *Actinobacteria* in marine sediments have been reported to vary from 0% to 17% [51]. Similar abundances have also been observed in culture-independent studies with the *Actinobacteria* component of bacterial communities accounting for 12.7% in forest soils, 21%–30% in soils of varying land use, 10% in Arctic deep sediments and 2%–4% in polar seawater [49]. Babalola *et al.* conducted a phylogenetic analysis of actinobacterial populations associated with Antarctic Dry Valley mineral soils using both traditional culture-based techniques and a metagenomic study [29]. Using actinobacterium and streptomycete-specific primers the majority of clones were related to uncultured *Pseudonocardia* and *Nocardioides* species and several rare *Actinobacteria* genera. However, their complementary culture-dependent work showed the presence of both *Nocardia* and *Pseudonocardia* in addition to *Streptomyces* species, which represented over 80% of the cultured isolates despite very low detection of this genus in the metagenomic study. This low detection of *Streptomyces* species using culture-independent techniques despite an abundance of cultured isolates is similar to observations in this study. It is interesting to note that the Babalola study used Actinobacteria-specific primers for the culture-independent diversity screening, which appeared not to improve detection of culturable actinomycetes (Babalola *et al.*, 2009) [29]. The inability to detect *Streptomyces* and other commonly cultured actinomycetes (e.g., *Micromonospora*) in culture-independent surveys of microbial diversity may due to insufficient sequencing depth, which readily detects OTUs present in high abundance, [5,6,8,31,52,53]. Underrepresentation of commonly cultured actinomycetes in 16S rRNA sequence libraries may also be due to inefficient cell lysis and DNA extraction within a complex substrate (e.g., sediment) and primer bias due to the high GC content of actinomycetes [54]. The apparent underrepresentation of readily cultured actinomycetes in culture-independent studies may be exacerbated by highly selective culture approaches, which target specific taxa and are biased towards the isolation of actinomycetes such as *Streptomyces*. Within this study improving DNA extraction efficiencies and exploring alternative 16S rDNA primer sets in combination with greater sequence coverage may have greatly enhanced detection of rare actinomycetes within the sediment bacterial communities [55]. 

### 2.3. Culture-Dependent Actinomycete Diversity

**Figure 5:**
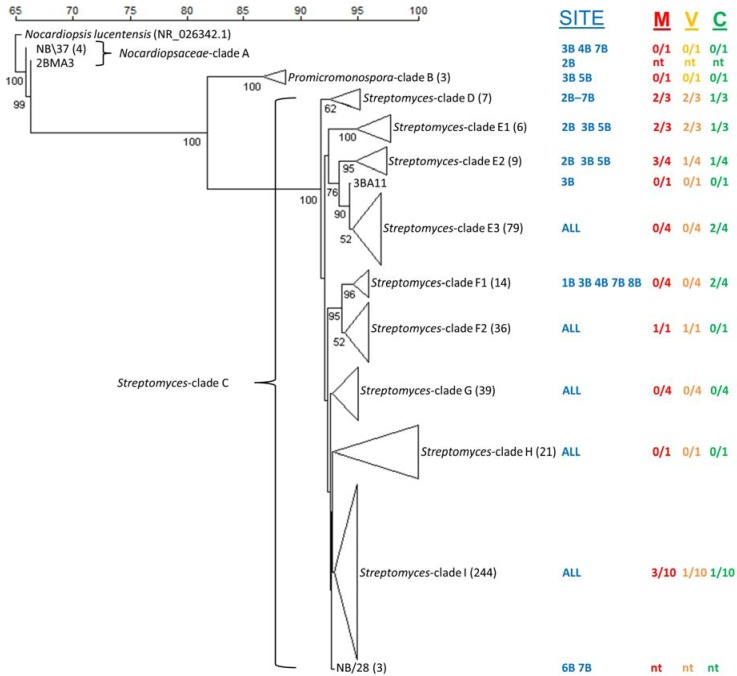
Neighbor-joining tree showing the phylogenetic relationship of cultured actinomycetes obtained from New Brunswick sediments. Numbers at nodes indicate the degree of bootstrap support (%) based on 1000 iterations, only values ≥50% are shown. The scale bar represents percent sequence similarity. The sequence of *Nocardiopsis lucentensis* (NR024362.1) was used as an out-group. Collection site (1B, 2B, 3B, 4B, 5B, 6B, 7B, 8B) and antimicrobial activity of crude fermentation extracts against the microbial pathogens MRSA (“M” colored red), VRE (“V” colored yellow) and *C. albicans* (“C” colored green) are displayed adjacent to each subclade. The antimicrobial activity results are represented by the first number being the number of active (>50% cell lysis) strains, with the second number (after the slash) the total number of isolates tested from that clade and ‘nt’ represents not tested. The tree branches within the phylogenetic tree correspond to an individual isolate, a consensus sequence or a clade comprised of both. The number in brackets after clade name is total number of isolates in each clade or contig group. Consensus sequences from sequence groups sharing >99% sequence similarities to other isolates are labeled with a “NB\” prefix. The numbers of isolates in these sequence groups are shown in brackets after the NB\ group number.

From our culture-independent bacterial community composition analysis, we knew that to access the culturable actinomycete diversity present in Bay of Fundy sediments a selective isolation strategy combining established highly taxa-selective media and pre-treatment methods should be used [27]. The aim was to selectively culture a diverse library of filamentous actinomycetes from sediment samples by inhibiting the growth of Gram-negative bacteria and selecting for the recovery of spore-forming, slow-growing actinomycetes [22,56]. A combination of three sediment pretreatments/plating techniques and five isolation media resulted in the isolation of 1500 actinomycetes. Isolates from each location were dereplicated based on morphological characteristics, resulting in the selection of 466 morphologically unique isolates for partial 16S rRNA gene sequencing. The number of isolates sequenced from each location was as follows: 1B-59, 2B-74, 3B-83, 4B-49, 5B-47, 6B-38, 7B-83 and 8B-33. Of the 466 morphologically unique isolates, the majority was isolated using the dry/stamp pretreatment (45%), compared to the dilute/heat method and direct plating method, which yielded 30% and 25% of isolates, respectively. The overgrowth of non-actinomycete bacteria using the direct plating method contributed to the lower number of isolates obtained using this approach. The majority of isolates were obtained on SC (152), RH (121) and PN (113) media, while substantially fewer actinomycetes were recovered on AC (61) and CH (19). Employing a 99% sequence identity cut-off, the resulting sequences were grouped into 39 OTUs consisting of multiple isolates and 49 singletons (Supplementary Tables S4 and S5). Grouping of sequences at a more conservative level (97% identity) resulted in 14 OTUs and 21 singletons. Isolates were identified by BLASTN comparison to sequences contained in GenBank and the phylogenetic relationships between the consensus sequences for OTUs (NB\1–NB\39), singletons and reference strains were analyzed by construction of a NJ tree (Figure 5).

Isolates from three major clades representing the families *Nocardiopsaceae* (Figure 5—Clade A), *Promicromonosporaceae* (Figure 5—Clade B) and *Streptomycetaceae* (Figure 5—Clade C). These clades are supported by high bootstrap values (99%–100%). Clade A (*Nocardiopsaceae*) comprises two OTUs consisting of one individual isolate 2BMA3 (site 2B, Black Beach) and OTU NB\37 that consists of four isolates obtained from sites 3B (Black Beach), 4B (Chance Harbour) and 7B (Pocologan) (Table S5). Clade B (*Promicromonosporaceae*) consists of two isolates, which share limited sequence identity (89%). These strains were isolated from sites 3B and 5B. The major *Streptomyces* clade is divided into six subclades (D–I) revealing fine-scale genetic variation among the cultured *Streptomyces* isolates. Some subclades are more supported (E1–F2) than others (G–I), reflected in their bootstrap values, suggesting that although all isolates are assigned to the *Streptomyces* genus, some subclades lack robust assignment within the order of the phylogenetic tree. Subclade D is fairly well supported by bootstrap analysis (62%) and consists of only seven isolates. Subclade E consists of three well-supported (bootstrap >90%) groups (E1, E2, E3) comprised of 81 isolates and 14 singletons. Subclade F consists of two supported groups (F1 96%, F2 52%) comprising 45 isolates distributed among nine sequence groups and five individual isolates. Subclade G consists of three OTUs and six individual isolates, collectively representing 33 isolates. Subclade H is the second smallest subclade with 16 isolates in two groups and five individual isolates while I is the largest subclade with 233 isolates distributed between 15 sequence groups and 14 individual isolates.

The taxonomic relationships of branches composed of the cultured isolates are supported by high-bootstrap values for nodes separating *Promicromonospora* species and *Nocardiopsis* species from *Streptomyces* species. However, within the *Streptomyces* clade several nodes are supported by lower bootstrap values, which is likely a result of the highly related nature of *Streptomyces* 16S rRNA gene sequences and the relatively short sequence lengths compared. Results show that the distribution of *Streptomyces* strains between the Bay of Fundy sediment samples are found to be generally equally distributed which could be a result of mixing due to the exceptionally high tides—although additional sampling would be required to support this conclusion. Furthermore, perhaps if more sensitive phylogenetic markers were used a greater taxonomic resolution could be obtained; in some cases it has been shown that multi-locus sequencing typing (MLST) using several housekeeping genes (involved in primary metabolism) have shown improved species resolution compared with 16S rRNA gene sequence comparisons for *Streptomyces* [57]. 

A few patterns could be discerned regarding the isolation of different taxonomic groups using the different isolation conditions employed in this study. *Nocardiopsis* strains (Clade A) were preferentially isolated using RH medium (four of five strains) and the DS pretreatment (three of five strains) while all *Promicromonospora* strains (Clade B) were isolated using SC medium and the DS pretreatment method. No trends were apparent within the *Streptomyces* subclades as very closely related *Streptomyces* were isolated on multiple media and using multiple pretreatments, demonstrating the ability of this genus to metabolize a diverse array of growth substrates. The majority of subclades (D–I) within the *Streptomyces* clade contain isolates, which originate from all isolation sites (Supplementary Table S5). Cultured actinomycetes do not cluster by geographic location as observed in the culture-independent microbial community analysis, this may suggest that there is little distinction of culturable actinomycetes between sites in the Bay of Fundy, as it appears that perhaps with sufficient sampling from a single location a similar diversity of culturable actinomycetes would be obtained from all sites. However, analysis of additional replicates within each site would be required to fully assess the affect of biogeography on the diversity of culturable actinomycetes. 

Within the phylogenetic tree (Figure 5), the *Streptomyces* clade is separated into six subclades according to the partial 16S rRNA gene sequences; however, as observed in other studies, the morphological variation did not agree with the phylogenetic grouping, which could be a result of the phenotypic plasticity of the isolated *Streptomyces* species [5]. All clades were composed of singletons and OTUs consisting of multiple isolates. All sites exhibit a high level of *Streptomyces* diversity, which may indicate a regional cosmopolitan distribution of *Streptomyces* species with a lack of biogeographical distribution. This biogeographical trend was observed by Wawrik *et al.* when studying actinomycete communities in soil, showing that actinomycete communities clustered by country of origin [58]. Wawrik *et al.* observed biogeography on a much broader scale, whereas the locations in this study were only separated by a few kilometers, thus actinomycete biogeography may operate on a larger scale [58]. Additionally, the actinomycete communities in this study looked quite homogenous between sites, while the overall bacterial communities observed by culture-independent methods exhibited distinct differences at the class level. This suggests that culturable actinomycete communities are not influenced by the same factors as the overall bacterial community. One explanation for this difference may be that the cultured actinomycetes are not metabolically active in the sediments, and are therefore not affected by the physiochemical conditions of the sediment. This could also explain the under-representation of actinomycetes in the culture-independent analysis because if actinomycetes were residing in the environment as spores, DNA from the spores may have been under-represented due to their resistance to lysis. The groups of multiple isolates (which contain isolates from multiple sediment locations) may represent more ubiquitous *Streptomyces* species within this intertidal area or potentially a result of land proximity; however, the ‘individual’ sequences may represent “rare” *Streptomyces* species only obtained by employing a thorough selective culture regime. However, in general many of the unique sequences are very closely related to sequence groups.

Comparison of culturable actinomycete diversity with overall bacterial diversity is problematic, due to the influence of a variety of selective culture conditions and environmental conditions. Previous studies have observed high levels of culturable actinomycete diversity from sediment. For example, almost two thirds of >1600 cultured isolates from Palau intertidal marine sediment, belonged to the class *Actinobacteria* [59]. Pathom-Aree *et al.* isolated diverse actinomycetes belonging to the genera *Dermacoccus, Kocuria*, *Micromonospora*, *Streptomyces*, *Tsukammurella* and *Williamsia* [6]. Whereas Jensen *et al.* observed significantly higher numbers of actinomycete colonies (6425) encompassing lower diversity [22]. However, lower diversity has also been observed. Chesapeake Bay sediments revealed actinomycetes accounted for only 0.15%–8.63% of cultured isolates [60]. In this study there was a lower diversity of actinomycetes relative to other studies [5,17,61] as reflected by the isolation of fewer families (for example, *Micromonopora* and *Nocardiopsis*), but not surprising as this study was highly selective for *Streptomyces*. Furthermore, it was previously observed that the frequency of *Micromonospora* decreases with increasing ocean depth [5]. Therefore, the result of relatively shallow sediments investigated in this study may explain the lack of cultured isolates belonging to this group.

To determine if the four sites investigated host unique culturable actinomycete communities an analysis of similarity (ANOSIM) was conducted using PRIMER. A data matrix was created by grouping the cultured actinomycete into OTUs based on 99% sequence identity (Supplementary Tables S4 and S5). This matrix was normalized by converting to percentage to account for the variation in the number of cultured isolates between sites. The R statistics generated from this analysis were 0.111, 0.083 and 0.667 for comparisons between Black Beach and Chance Harbour, Black Beach and (7B and 8B) and Chance Harbour and (7B and 8B) respectively. 7B and 8B could not be compared to each other due to lack of replicates and were therefore combined as an artificial location for this analysis. With *R* statistic values far from 1 and *p* values between 10% and 40%, the results reflect observations made from the phylogenetic tree, that the different sites do not contain unique culturable actinomycete communities; however, further sampling would be required to confirm these trends.

Canonical correspondence analysis (CCA) was preformed to statistically assess if the sediment parameters influenced the culture-dependent community composition. The community composition was based on grouping of cultured isolates at 99% sequence similarity. Due to the large data set, again the CCA analysis was split into three groups of sediment variables to enable each variable to be tested for significance. Group one (Supplementary Figure S4: % Organic Matter, pH, P_2_O_2_, K_2_O, Ca and Mg), group two (Supplementary Figure S5: B, Cu, Fe, Na, Zn, S) and group three (Supplementary Figure S6: sediment particle size: size 1 (<0.6 mm), size 2 (0.6–1 mm), size 3 (1–2 mm) and size 4 (>2 mm)). The correlation between culturable actinomycete community composition and the physiochemical variables were not statistically significant as p values for all CCAs greatly exceed the significance level of 0.05 (Group 1, *p* = 0.498; Group 2, *p* = 0.551; Group 3, *p* = 0.185). Sediment particle size (group 3) appeared to have the greatest (albeit not statistically significant) influence on the cultured actinomycete community, with the large particle size 4 (>2 mm) correlated with site 8B (Mispec Bay) and the small particles (size 1: <0.6 mm and size 2: 0.6–1 mm) correlated with sites 1B, 2B and 7B with the remaining three sites correlated with sediment size 3 (1–2 mm). It would be interesting to conduct further repetition of sampling incorporating temporal and spatial patterns to further investigate the effect of particle size on actinomycete community composition.

To investigate the potential of several of the isolated actinomycetes to produce bioactive secondary metabolites, 35 isolates, representing strains from each clade were fermented and the corresponding organic extracts tested for antimicrobial activity against five pathogens. Overall, 43% of the strains tested exhibited bioactivity (*i.e*., >50% growth inhibition) against one or more pathogens. Activity was observed against MRSA, VRE and *C. albicans*; however, none of the extracts inhibited the growth of the Gram-negative pathogens *P. vulgaris* and *P. aeruginosa*. Anti-MRSA activity was the most prevalent with 28% of strains inhibiting the growth of this organism, followed by anti-*C. albicans* (23%) and anti-VRE (17%) activity. Of strains exhibiting antimicrobial activity, the majority (60%) produced antimicrobial activity against a single pathogen, while the remainder exhibited activity against two or more pathogens simultaneously, suggesting the production of a single metabolite with broad-spectrum activity or the production of multiple metabolites with narrow activity spectrums. 

Three *Streptomyces* strains exhibiting antimicrobial activity from clades D (4BB3; OTU NB/38), E (4BB9) and F (7BF3; OTU NB/3) were fermented on a larger scale to determine the natural products responsible for the observed antimicrobial activity. This resulted in identification of several previously reported natural products based on high-resolution mass spectrometry profiling of crude extracts. Fermentation extracts of *Streptomyces* sp. 4BB3 inhibited the growth of MRSA, VRE and *C. albicans*. The major metabolite in this extract was identified as staurosporine based on HRMS and NMR analyses. Staurosporine may be responsible for the observed anti-*C. albicans* activity, however, as this compound does not exhibit antibacterial activity, other minor metabolites in the extract must be responsible for the VRE and MRSA activity [62]. PM050463 (an α-pyridone antibiotic) was identified as a major metabolite in 4BB9 extracts, which inhibited the growth of MRSA, VRE and *C. albicans* [63] Antibiotics belonging to the α-pyridone class possess diverse antimicrobial activities, thus, PM050463 is likely responsible for the observed activity. Novobiocin was isolated from 7BF3 and identified by HRMS and NMR. Novobiocin is responsible for the observed activity against MRSA, VRE and *C. albicans*. Novobiocin has been reported to possess a wide range of antimicrobial activities thus the activity profile of 7BF3 is consistent with reported activity. 

Although species-specific secondary metabolite production has been observed for marine *Salinispora* species, this might not be the case for *Streptomyces* [22,64]. Several studies have found that *Streptomyces* strains with 16S sequence identities >99% frequently produce different metabolites, leading to the conclusion that the commonly employed 3% difference in 16S rRNA sequence likely does not accurately delineate *Streptomyces* species [64]. Perhaps a factor of the highly conserved nature of the 16S rRNA gene and complex nature of *Streptomyces* taxonomy, it has been suggested that full-length 16S rRNA values closer to 99% would serve as a more realistic level to achieve species level discrimination [53]. Ninety-nine percent sequence identity was used to group OTUs in this study to describe the taxonomic diversity within this highly conserved genus. If 99% identity of 16S rRNA genes was accepted as a species cut-off within the *Streptomyces* genus, this study would have revealed several new *Streptomyces* species isolated from Atlantic Canada. 

Many studies have shown the exceptional potential of *Streptomyces* species for the production of bioactive compounds [1,5,65,66,67]. The subset of strains tested in this study demonstrated considerable antimicrobial activity. Forty-three percent of the 35 strains tested inhibited the growth of one or more of the microbial pathogens (Figure 5). The frequency of antimicrobial activity is similar to what was observed in *Streptomyces-*like strains isolated from the Trondheim Fjord in Norway [5], which ranged from 34% to 47% and 23% to 42% activity against *Micrococcus luteus* and *C. albicans*, respectively. The high frequency of activity observed in the small subset of strains highlights the great potential of the culture library for the production of bioactive natural products as a resource for drug discovery. Preliminary investigations identified several previously identified compounds from bioactive fermentation extracts, which is a common challenge in natural product research [4,51]. Future efforts will more fully explore the natural product repertoire of the members of this culture library using statics-guided analysis of LC-HRMS profiles of fermentation extracts [68]. Given that most actinomycete genomes contain 10–20 secondary metabolite gene clusters [69,70]. The application of highly sensitive chemical screening techniques will undoubtedly lead to the discovery of novel metabolites, some of which may have therapeutic potential.

## 3. Experimental Section

### 3.1. Sample Collection and Processing

Eight sediment samples were collected in August 2009 from intertidal zones of the Bay of Fundy, New Brunswick, Canada. Samples were collected from four locations where tides vary from 9–11 m [71]. Three black rocky sediment samples were collected at Black Beach (45°0′55.00′′, −66°13′46.00′′) and are named 1B, 2B and 3B representing low, mid- and high tide levels, respectively. Three sandy sediment samples were collected at Chance Harbour (44°7′53.50′′, −66°20′42.46′′) and are named 4B, 5B and 6B representing low, mid- and high tide levels, respectively. One mud sample (7B) was collected from the Pocologan mud flat (45°7′24.13′′, −66°34′47.39′′) and one rocky sediment sample (8B) was collected from Mispec Bay at low tide (approximately 2 m underwater at high tide) (45°13′11.00′′, −65°57′0.00′′). Samples were collected aseptically using a sterile scoopula and 50 mL conical tube. Samples were transported to the laboratory on ice (≤4 h), after which a portion (~2 g) of each sample was immediately archived for future DNA extraction and stored at −80 °C and the remainder was processed for actinomycete culturable diversity studies (see Actinomycete Isolation methods). For sediment analysis, larger samples (*ca*. 200 g) were collected in a plastic container, excess seawater decanted, and stored at −80 °C.

### 3.2. Sediment Analysis

Chemical analyses of sediment samples were conducted at the Prince Edward Island Department of Agriculture and Forestry Soil and Feed Testing Laboratory (Charlottetown, Canada). Analysis included organic matter content [72] pH, determination of phosphate (P_2_O_5_), potash (K_2_O), calcium, magnesium, boron, copper, zinc, sulphur, manganese, iron andsodium levels [73]. To determine the distribution of particle sizes in sediment a known weight of each sediment sample was passed through a series of soil sieves (2 mm, 1 mm, 0.6 mm) (Canadian standard sieve series, ON) and the weight of sediments retained by each sieve was recorded. 

### 3.3. Isolation of Sediment Metagenomic DNA

Approximately 1.25 g of thawed sediment was washed twice with sterile 1% (w/v) NaCl (500 µL) to remove water-associated bacteria. DNA was extracted from approximately 500 mg of each sediment sample using the Fast DNA Spin Kit for Soils according to the manufacturer’s recommendations (MP Biomedicals, Solon, OH, USA) and stored at −20 °C. Concentration and integrity of isolated DNA was determined by UV spectroscopy and agarose gel electrophoresis (1% agarose, 1× Tris-acetate-EDTA buffer stained with ethidium bromide) [74]. 

### 3.4. 16S rRNA Amplicon Pyrosequencing and Sequence Analysis

Bacterial diversity was assessed by pyrosequencing of 16S rRNA amplicons generated from sediment metagenomic DNA. Bacterial tag-encoded FLX amplicon pyrosequencing (bTEFAP) of metagenomic 16S rRNA amplicons was performed by Research and Testing Laboratory (RTL; Lubbock, TX, USA) as previously described [75] based upon RTL protocols [76]. bTEFAP utilizes Titanium reagents and procedures with a one step PCR reaction containing a mixture of both HotStart and HotStart high fidelity *Taq* polymerases and amplicons originating from the 27F region numbered in relation to *E. coli* rRNA [77]. 

To reduce the effect of sequencing artifacts on downstream data analysis, sequence data were processed using mothur version 1.32.0 [78] according to published recommendations [79,80]. Briefly, sequence data were de-noised using the mothur implementation of PyroNoise (shhh.flows), and sequences were removed if they contained homopolymers greater than 8 bp in length, ambiguous bases, more than one mismatch to barcode sequences and more than two mismatches to the forward primer sequence, or were shorter than 200 bp. Sequences passing these quality control criteria were aligned in mothur using the Silva reference alignment obtained from the mothur website [81]. Alignments were screened to ensure the sequences overlapped in the same alignment space. Alignments were filtered to remove gaps, and then sequences were preclustered using a “diffs” setting of 2. Chimeras were identified using UCHIME and removed from the analysis. Sequences were classified using the mothur Bayesian classifier (80% confidence) utilizing the mothur-formatted version of the Ribosomal Database Project (RDP) training set (v.9) [82]. Sequences classified as chloroplasts, mitochondria, or “unknown” (sequences not classified at the kingdom level) were removed from the analysis. OTUs were identified as described previously [80]. Observed richness, estimated richness (Chao1), sampling coverage (*C*), and the Shannon diversity (*H*’) and evenness (*E*) indices were calculated using mothur. Sequence information is available through NCBI sequence read archive (accession numbers: SRX218538-SRX218545).

### 3.5. Cluster Analysis of (a) Culture-Independent Bacterial Communities (b) Comparison of Sediment Chemical Composition

The species level (*D =* 0.03) OTU data matrix was obtained using mothur and then entered into Microsoft Excel (2007). Raw OTU abundance was converted to percent abundance for each site and the percent normalized data matrix was imported into PRIMER 5 (Primer-E Ltd., Plymouth, UK) [82,83]. A Bray-Curtis similarity matrix was generated applying a square root transformation and hierarchical clustering was performed using complete linkage to compare the composition of species level bacterial communities. An identical analysis was conducted on a data matrix consisting of sediment composition data. 

### 3.6. Analysis of Similarities (ANOSIM) of (a) Culture-Independent Bacterial Communities (b) Culturable Actinomycete Diversity by Location

Using PRIMER 5 (Primer-E Ltd., Plymouth, UK), previously generated transformed-Bray-Curtis similarity matrixes (see cluster analysis methods) were used to preform ANOSIM statistical pairwise comparison by location. OTU matrixes were normalized to percentage to account for varying sample size and were based on (a) mothur analysis of 16S rDNA amplicon sequence libraries and (b) cultured actinomycete grouping by 99% sequence identity, where singletons were defined as individual OTUs. Location groups for both analyzes were Black Beach (samples 1B, 2B and 3B), Chance Harbour (samples 4B, 5B and 6B) and an artificial location group (samples 7B and 8B). 7B and 8B were grouped together as there are no replicate samples from these locations.

### 3.7. Canonical-Correspondence Analysis (CCA) Testing the Influence of Sediment Parameters on (a) Culture-Independent Bacterial Communities (b) Culturable Actinomycete Diversity by Location

CCA analysis was performed using XLSTAT-ADA (Addinsoft, USA) to find the association between two sets of variables using 1000 permutations and a 5% significance level. Variable one was sediment composition and the second variable was (a) a percent normalized species level OTU data matrix (see cluster analysis methods) and (b) cultured actinomycete diversity (grouping by 99% sequence identity where singletons were defined as individual OTUs). Due to the large data set, CCA analysis was split into three groups of sediment variables to enable each variable to be tested for significance. Group one (% Organic Matter, pH, P_2_O_2_, K_2_O, Ca and Mg), group two (B, Cu, Fe, Na, Zn, S) and group three (sediment particle size: size 1 (<0.6 mm), size 2 (0.6–1 mm), size 3 (1–2 mm) and size 4 (>2 mm)).

### 3.8. Actinomycete Culturable Diversity

A combination of sediment pre-treatments, plating techniques and selective isolation media were employed to selectively isolate mycelia-forming actinomycetes. The dry/stamp (DS) method involved drying sediment overnight in a laminar flow hood. Dried sediments were subsequently stamped onto the surface of agar plates using a sterile foam plug (2 cm diameter) eight times to create a serial dilution effect. The dilute/heat (DH) method involved diluting 1 mL of wet sediment with 10 mL sterile seawater and then heating the diluted sediment to 55 °C for 6 min. A 200 µL aliquot was used to inoculate each agar plate [22]. The direct plating (DP) method consisted of spreading 1 g of sediment directly on to each agar plate. For each pretreatment and plating combination, five isolation media were used: Starch Casein Nitrate agar (SC), Raffinose-Histidine agar (RH) [8], Chitin Low Nutrient agar (CH) [84], Phosphate-Nitrate agar (PN) [85] and Actinomycete Isolation agar (AC) [86]. All media were prepared with filtered natural seawater (collect from the Southern coast of Prince Edward Island, Canada) and sterilized by autoclaving. To reduce the growth of fungi and non-actinomycete bacteria, media were supplemented with nystatin (AC—10 µg mL^−1^; RH, CH, SC, PN—50 µg mL^−1^); cycloheximide (AC—50 µg mL^−1^; RH, CH—100 µg mL^−1^) and nalidixic acid (SC, PN—10 µg mL^−1^). Four replicates of each pretreatment (DH, DS, DP) and media (RH, SC, AC, CH, PN) combination were plated resulting in 60 isolation plates per sediment and 480 plates in total.

Plates were monitored for the emergence of colonies exhibiting morphology typical of filamentous actinomycetes (leathery substrate mycelia and formation of aerial mycelia and spores) for three months. Colonies exhibiting the targeted morphology were chosen for purification and initially subcultured onto the same media from which they were isolated, but prepared with 18 g L^−1^ Instant Ocean^®^ (IO; Spectrum Brands) and Milli-Q water to replace seawater. Isolates were recursively subcultured onto International *Streptomyces* Project Medium 3 (ISP-3) [86,87] supplemented with 18 g L^−1^ IO until pure. Isolates were dereplicated within sample location based on morphological characteristics (colour and texture of both aerial and substrate mycelia, production of soluble pigments and presence/absence/color of spores). Cultures were archived as spore suspensions or vegetative mycelia in 20%–25% glycerol (v/v) and stored at −80 °C.

### 3.9. Identification of Bacterial Isolates

Axenic isolates were cultured in 7 mL of International *Streptomyces* Project Medium 2 (ISP2) [87] prepared with 18 g L^−1^ IO and Milli-Q water at 30 °C and shaken at 200 rpm. Genomic DNA was extracted according to established protocols [88]. Amplification of 16S rRNA genes was conducted in 50 µL volumes and consisted of the following: EconoTaq^®^ PLUS GREEN 2X Master Mix (25 µL) (Lucigen, Middleton, WI, USA), 0.5 µM each of the primers pA (5′-AGAGTTTGATCCTGGCTCAG) and pH (5′-AAGGAGGTGATCCAGCC) [89] and genomic DNA (2.5 µL of a 10^−1^ dilution). Thermal cycling parameters consisted of initial denaturation at 95 °C for 1 min, 30 cycles of 95 °C for 1 min, 57.6 °C for 1 min 30 s and 72 °C for 2 min followed by a final extension at 72 °C for 5 min. A negative control, which lacked template DNA, was included in each set of PCR reactions. Amplification was evaluated by agarose gel electrophoresis as described earlier.

Partial sequencing of 16S rRNA amplicons was performed by Genome Quebec (Montreal, Canada) or Eurofins MWG Operon (Huntsville, AL, USA) using the 16S530R primer 5′-GTATTACCGCGGCTGCTGG [90]. In some cases full-length 16S rRNA gene sequencing was performed using the additional primers 16S936R (5′-GGGGTTATGCCTGAGCAGTTTG), 16S1527R (5′-AAGGAGGTGATCCAGCC), 16S514F (5′-GTGCCAGCASCCGCGG) and 16S1114F (5′-GCAACGAGCGCAACCC) [59]. 

### 3.10. Phylogenetic Analysis of Cultured Actinomycetes

Sequences (~530 bp) were analyzed, edited and grouped using the Contig Express application within the Vector NTI version 10.3 software package (Invitrogen, Carlsbad, CA, USA). Sequences were compared to sequences within the NCBI database [91] using the Basic Local Alignment Search Tool (BLASTN) [92]. Phylogenetic analysis of partial 16S rRNA gene sequences was conducted using Bionumerics (Version 6.1, Applied Maths, Austin, TX, USA). Following multiple sequence alignment [93] phylogenetic trees were inferred using the neighbour-joining (NJ) algorithm using default settings [94]. The robustness of the resulting tree was evaluated by bootstrap analysis of NJ data based on 1000 resamplings using the Bionumerics software [95]. The partial 16S rRNA gene sequences of all 49 individual isolates and one representative isolate of all 39 sequence groups (at 99% sequence identity) were deposited in the GenBank nucleotide sequence database under the accession numbers KF646000 to KF646087.

### 3.11. Fermentation and Extraction

ISP2 medium prepared with 18 g L^−1^ IO and Milli-Q water was dispensed (10 mL) into culture tubes (150 × 25 mm) containing 3–5 soda lime glass beads and sterilized by autoclaving (121 °C for 30 min). Seed cultures were inoculated with fresh growth from an agar plate (ISP2 solidified with 18 g L^−1^ agar) and incubated at 30 °C and 200 rpm for 72 h. IO was not included in seed and fermentation media because all strains exhibited robust growth on media lacking salt. Omitting IO also enhanced subsequent chemical analysis of fermentation extracts by reducing the levels of co-extracted salt present in extracts. Fermentations (10 mL ISP2) were inoculated with 0.5 mL of the seed culture and incubated under identical conditions for seven days. 

Fermentations were extracted with 10 mL ethyl acetate by rapid agitation (200 rpm) at room temperature for one hour. Extracts were dried *in vacuo* then partitioned between 8:2 acetonitrile:water and hexanes (10 mL each). Extracts were dried *in vacuo* and the weights recorded.

### 3.12. Antimicrobial Testing

Microbroth assays were conducted in 96-well plates according to the Clinical Laboratory Standards Institute testing standards (Methods for Dilution Antimicrobial Susceptibility Tests for Bacteria that Grow Aerobically; Approved Standard, Sixth Ed, M7-A6 Volume 23, number 2). Extracts were evaluated for antibiotic activity against methicillin-resistant *Staphylococcus aureus* ATCC 33591 (MRSA), vancomycin-resistant *Enterococcus faecium* 379 (VRE), *Pseudomonas aeruginosa* ATCC 14210, *Proteus vulgaris* ATCC 12454, and *Candida albicans* ATCC 14035. Extracts were dissolved in sterile 20% dimethyl sulfoxide (DMSO) and tested at a final concentration of 100 µg/mL. Extracts were considered active if they exhibited >50% growth inhibition.

### 3.13. Chemical Analysis of Fermentation Extracts

Fermentation extracts were analyzed by ultrahigh performance liquid chromatography-high-resolution mass spectrometry (UPLC-HRMS) using an Orbitrap Exactive (Thermo Fisher Scientific, Mississauga, ON, Canada) HPLC system with Kinetex 1.7 µm C18 100 Å 50 × 2.1 mm column (Phenomenex, Torrance, CA, USA). A linear gradient from 95% H_2_O/0.1% formic acid (solvent A) and 5% acetonitrile/0.1% formic acid (solvent B) to 100% solvent B over 5 min followed by a hold of 100% solvent B for 3 min was used with a flow rate of 400 µL/min. Eluent was detected by ESI-HRMS monitoring *m/z* 200–2000 in positive mode, evaporative light scattering detector (ELSD; Sedex) and PDA (200–600 nm).

Compounds were purified using a Waters HPLC system with a semi preparative Gemini 5 µm C18 110Å 250 × 10 mm column (Phenomenex, Torrance, CA, USA) and eluate was monitored by ELSD and PDA (254 and 280 nm). Compounds were identified using a NMR spectrometer (Bruker Avance 600 MHz). Spectral data from 1D and 2D NMR experiments and UPLC-HRMS analysis were compared to literature data to confirm the identity of known compounds.

## 4. Conclusions

This study demonstrates the success of investigating novel environments using bTEFAP to build knowledge of bacterial community structure, to in turn enable selective cultivation strategies to be designed allowing for prioritization of bacterially diverse locations for further investigation. All eight sediment samples from the Bay of Fundy contained diverse bacterial communities. Using pyrosequencing and diversity analysis, it was found that overall bacterial community structure was affected by sediment physiochemical properties, therefore, sites within the same location had similar microbial communities and distinct bacterial communities were observed between sites. 

An exceptional number of culturable actinomycete species were isolated, in particular *Streptomyces* strains. Biogeography did not appear to influence actinomycete populations. The library of cultured *Streptomyces* generated from this research provides bioprospecting information and paves the way for future *Streptomyces* research in these under-studied habitats. Insight into the relationships between actinomycete diversity, overall bacteria community structure and environmental factors is fundamental for understanding the ecology of this well-studied family in marine environments. The vast number of *Actinobacteria* isolated from New Brunswick during this research, and their unrivalled success rate for producing bioactive secondary metabolites, combined with novel bioprospecting locations, highlights the importance of New Brunswick as a diverse source of secondary metabolites with widespread biotechnological potential. 

## Supplementary Files

Supplementary File 1 Supplementary Information (PDF, 1169 KB)
