# Supplementary material for: Bioprospecting from Marine Sediments of New Brunswick, Canada: Exploring the Relationship between Total Bacterial Diversity and Actinobacteria Diversity
Source: Mar Drugs. 2014 Feb 13;12(2):899–925. doi: 10.3390/md12020899 (PMC3944522; doi:10.3390/md12020899)
Supplement: Supplementary File 1 — Supplementary Information (PDF, 1169 KB) [file marinedrugs-12-00899-s001.pdf]

Supplementary Information

Table S1. Phylum level composition of bacterial communities in eight New Brunswick sediments.

**Phylum**	**IB ***	**IB%**	**2B***	**2B%**	**3B ***	**3B%**	**4B ***	**4B%**	**5B ***	**5B%**	**6B ***	**6B%**	**7B ***	**7B%**	**8B ***	**8B%**
Acidobacteria	270	4.0	248	5.3	383	6.4	81	1.0	63	1.4	73	1.4			474	7.5
Actinobacteria	542	8.0	111	2.4	181	3.0									168	2.7
Bacteroidetes	1882	27.7	1133	24.4	1196	20.1	1645	20.9	879	20.1	990	18.7	1450	39.5	2012	32.0
Chlorobi									54	1.2	66	1.2				
Planctomycetes	88	1.3														
Proteobacteria	2284	33.6	1851	39.9	2468	41.4	4349	55.4	2430	55.4	3020	56.9	1523	41.5	2510	39.9
Verrucomicrobia	307	4.5											133	3.6	65	1.0
Unclassified Bacteria	1367	20.1	1205	26.0	1581	26.5	1474	18.8	824	18.8	1058	19.9	487	13.3	926	14.7
"Rare Phyla"	51	0.8	95	2.0	148	2.5	223	2.8	133	3.0	99	1.9	79	2.2	128	2.0
Total	6791	100.0	4643	100.0	5957	100.0	7856	100.0	4383	100.0	5306	100.0	3672	100.0	6283	100.0

* Denotes number of sequences.

Table S2. Number of sequences represented in the "Rare Phyla" group presented in Table S1.

**Phylum**	**IB**	**2B**	**3B**	**4B**	**5B**	**6B**	**7B**	**8B**
Acidobacteria							5	
Actinobacteria				62	39	33	13	
Armatimonadetes	1							9
Chlorobi	13	2	5					5
Chloroflexi	3	4	4	12	27	8	6	20
Fusobacteria			3	1	1		1	
Gemmatimonadetes	9		1		1			9
Lentisphaerae	4	2		2	2		12	4
Nitrospira	5	1	22				2	36
Planctomycetes		37	55	52	18	21	9	28
Spirochaetes				2			1	
Verrucomicrobia		46	51	73	33	26		

Table S2. *Cont.*

Cyanobacteriachloroplast							7	
Firmicutes	12	2	4	16	9	9	22	10
OD1	2		1					3
TM7	2	1	1					4
WS3			1	3	3	2	1	
Total	51	95	148	223	133	99	79	128

Table S3. Family level taxonomic composition with in the Order Actinomycetales.

**FamilywithintheOrder *Actinomycetales***	**1B ***	**1B%**	**2B ***	**2B%**	**3B ***	**3B%**	**4B ***	**4B%**	**5B ***	**5B%**	**6B ***	**6B%**	**7B ***	**7B%**	**8B ***	**8B%**
Cellulomonadaceae	4.0	1.0	1.0	2.0			6.0	40.0								
Demequinaceae	17.0	4.2	2.0	4.0											1.0	2.3
Geodermatophilaceae	4.0	1.0	1.0	2.0	4.0	3.8									2.0	4.5
Intrasporangiaceae	10.0	2.5	1.0	2.0											3.0	6.8
Kineosporiaceae															2.0	4.5
Microbacteriaceae	116.0	28.9	3.0	6.0	14.0	13.2									6.0	13.6
Micrococcaceae	7.0	1.7	3.0	6.0	6.0	5.7	1.0	6.7							1.0	2.3
Nakamurellaceae			1.0	2.0												
Nocardioidaceae	122.0	30.3	9.0	18.0	31.0	29.2	6.0	40.0							16.0	36.4
Propionibacteriaceae	5.0	1.2			2.0	1.9										
Pseudonocardiaceae	7.0	1.7														
Streptomycetaceae	6.0	1.5														
UnclassifiedActinomycetales	100.0	24.9	29.0	58.0	47.0	44.3	2.0	13.3	5.0	100.0	3.0	100.0	1.0	100.0	13.0	29.5
"RareFamily"	4.0[a]	1.0			2.0[b]	1.9										
Total	402.0	100.0	50.0	100.0	106.0	100.0	15.0	100.0	5.0	100.0	3.0	100.0	1.0	100.0	44.0	100.0

* Denotes number of sequences; [a] 1B rare family: 1 Micromonosporaceae, 1 Kineosporiaceae, 1 Nakamurellaceae, 1 Nocardiaceae; [b] 3B rare family: 1 Pseudonocardiaceae, 1 Intrasporangiaceae.

Table S4. Closest GenBank match of sequence groups (>99% sequence identity) and unique isolates. * Isolate name in brackets indicates the reference isolate from each sequence group that was deposited in GenBank.

Sequence Group/Strain Designation	Number of strains	Sequence length (bp)	Closest GenBank Match (Accession number)	% Identity	% Query coverage	Genbank accession number *
1	123	508	Streptomyces griseus strain ABRIINW 13 16S ribosomal RNA gene, partial sequence (HQ597007.1)	100	89	KF646049 (6BA6)
2	2	461	Streptomyces sp. 220297 16S ribosomal RNA gene, partial sequence (GU130099.1)	99	98	KF646050 (1BA12)
3	5	462	Streptomyces caeruleus strain NRRL 2449 16S ribosomal RNA gene, partial sequence (EF178698.1)	98	99	KF646051 (3BD4)
4	19	458	Streptomyces sp. SXY49 16S ribosomal RNA gene, partial sequence (GU045532.1)	99	98	KF646052 (4BD8)
5	4	454	Streptomyces azureus strain NRRL B-2655 16S ribosomal RNA gene, partial sequence (NR_044136.1)	99	99	KF646053 (1BB9)
6	53	465	Streptomyces albidoflavus strain HD-109 16S ribosomal RNA gene, partial sequence (EF620361.1)	99	97	KF646054 (5BC6)
7	7	459	Streptomyces sp. SXY124 16S ribosomal RNA gene, partial sequence (GU045539.1)	99	98	KF646055 (7BF5)
8	13	458	Streptomyces drozdowiczii partial 16S rRNA gene, isolate PhyCEm-1349 (AM921646.1)	99	98	KF646056 (3BC11)
9	20	454	Streptomyces baarnensis strain NRRL B-1902 16S ribosomal RNA gene, partial sequence (EF178688.1)	99	100	KF646059 (2BA6)
10	7	451	Streptomyces sp. SirexAA-E, complete genome (CP002993.1)	99	99	KF646079 (6BC12)
11	2	844	Streptomyces sp. BK178 partial 16S rRNA gene, strain BK178 (FR692110.1)	99	99	KF646064 (3BG7)
12	7	453	Streptomyces albidoflavus strain HD-109 16S ribosomal RNA gene, partial sequence (EF620361.1)	99	99	KF646067 (4BC6)
13	11	459	Streptomyces sp. SirexAA-E, complete genome (CP002993.1)	99	97	KF646085 (7BF12)
14	2	455	Streptomyces lavendulae strain NRRL B-1230 16S ribosomal RNA gene, partial sequence (HQ426713.1)	99	100	KF646063 (3BG2)
15	5	449	Streptomyces sp. HB108 16S ribosomal RNA gene, partial sequence (GU213496.1)	99	98	KF646070 (5BA5)
16	5	454	Streptomyces flavogriseus partial 16S rRNA gene, type strain CBS 101.34T (AJ494864.1)	99	98	KF646061 (2BG3)
17	4	449	Streptomyces drozdowiczii partial 16S rRNA gene, isolate PhyCEm-1258 (AM921645.1)	100	99	KF646075 (5BC2)
18	3	453	Streptomyces sp. AKB-2008-ET11 partial 16S rRNA gene, strain AKB-2008-ET11 (AM988883.1)	99	99	KF646078 (6BC5)
19	12	458	Streptomyces sp. HBUM80670 16S ribosomal RNA gene, partial sequence (EU119202.1)	99	98	KF646076 (6BA9)
20	5	448	Streptomyces drozdowiczii partial 16S rRNA gene, isolate PhyCEm-1258 (AM921645.1)	99	100	KF646065 (4BC2)

Table S4. *Cont.*

21	2	875	Streptomyces caeruleus strain NRRL 2449 16S ribosomal RNA gene, partial sequence (EF178698.1)	98	99	KF646068 (4BD5)
22	2	446	Streptomyces sp. DSM 21069 16S ribosomal RNA gene, partial sequence (FJ872524.1)	99	100	KF646069 (4BD7)
23	6	454	Streptomyces sp. ME02-6978.2d 16S ribosomal RNA gene, complete sequence (EU080941.1)	99	98	KF646060 (2BB1)
24	3	449	Streptomyces phaeochromogenes strain NRRL B-2031 16S ribosomal RNA gene, partial sequence	98	100	KF646082 (7BE9)
25	2	418	Streptomyces peucetius gene for 16S rRNA, partial sequence (AB249907.1)	99	100	KF646071 (5BA11)
26	11	451	Streptomyces sp. HB116 16S ribosomal RNA gene, partial sequence (GU213500.1)	100	99	KF646083 (7BE12)
27	2	455	Streptomyces sp. SirexAA-E, complete genome (CP002993.1)	99	98	KF646074 (5BC1)
28	3	452	Streptomyces sp. VTT E-99-1326 (A4) 16S ribosomal RNA gene, partial sequence (AF429390.1)	100	99	KF646077 (6BB7)
29	14	456	Streptomyces bottropensis strain cfcc3173 16S ribosomal RNA gene, partial sequence (GQ258687.1)	99	99	KF646058 (2BA2)
30	2	447	Streptomyces globosus strain 12620-1 16S ribosomal RNA gene, partial sequence (EF371433.1)	99	100	KF646081 (7BE6)
31	6	461	Streptomyces sp. AKB-2008-ET11 partial 16S rRNA gene, strain AKB-2008-ET11 (AM988883.1)	99	98	KF646087 (8BC10)
32	4	450	Streptomyces caeruleus strain NRRL 2449 16S ribosomal RNA gene, partial sequence (EF178698.1)	99	98	KF646072 (5BB1)
33	6	517	Streptomyces sp. 219840 16S ribosomal RNA gene, partial sequence (HQ992732.1)	99	87	KF646086 (8BA6)
34	7	456	Streptomyces sp. 219840 16S ribosomal RNA gene, partial sequence (HQ992732.1)	99	99	KF646057 (1BD9)
35	9	463	Streptomyces griseus strain ABRIINW 13 16S ribosomal RNA gene, partial sequence (HQ597007.1)	99	98	KF646062 (3BD11)
36	20	544	Streptomyces sp. ME01-21h 16S ribosomal RNA gene, complete sequence (EU080953.1)	100	82	KF646073 (5BB10)
37	4	439	Nocardiopsis dassonvillei subsp. dassonvillei DSM 43111 chromosome 2, complete sequence (CP002041.1)	99	100	KF646066 (4BC3)
38	3	450	Streptomyces peucetius strain JCM 9920 16S ribosomal RNA, partial sequence >dbj|AB045887.1| Streptomyces peucetius gene for 16S Rrna	99	96	KF646080 (7BB10)
39	2	510	Streptomyces flavogriseus ATCC 33331, complete genome (CP002475.1)	100	86	KF646084 (7BF2)
1BA3	1	452	Streptomyces tritolerans strain DAS 165 16S ribosomal RNA gene, partial sequence (DQ345779.2)	99	100	KF646009
1BB1	1	1515	Streptomyces sp. ME01-21h 16S ribosomal RNA gene, complete sequence (EU080953.1)	99	100	KF646008
1BC7	1	450	Streptomyces sp. HB110 16S ribosomal RNA gene, partial sequence (GU213497.1)	99	99	KF646001

Table S4. *Cont.*

1BD6	1	461	Streptomyces sp. AKB-2008-ET11 partial 16S rRNA gene, strain AKB-2008-ET11 (AM988883.1)	99	98	KF646002
1BE1	1	438	Streptomyces griseus strain ABRIINW 13 16S ribosomal RNA gene, partial sequence (HQ597007.1)	98	100	KF646003
1BE3	1	461	Streptomyces albidoflavus strain HD-109 16S ribosomal RNA gene, partial sequence (EF620361.1)	98	98	KF646004
1BE5	1	435	Streptomyces sp. HB184 16S ribosomal RNA gene, partial sequence (GQ863916.1)	99	100	KF646005
1BE11	1	455	Streptomyces sp. ME01-21h 16S ribosomal RNA gene, complete sequence (EU080953.1)	99	99	KF646006
2BA12	1	456	Streptomyces bikiniensis strain DSM 40581 16S ribosomal RNA, partial (NR_026177.1)	98	98	KF646007
2BB11	1	446	Streptomyces albidoflavus strain HD-109 16S ribosomal RNA gene, partial (EF620361.1)	99	98	KF646010
2BC11	1	443	Streptomyces badius strain NRRL B-2567 16S ribosomal RNA, partial sequence (NR_043350.1)	98	97	KF646011
2BD2	1	318	Streptomyces sp. JJB-6 16S ribosomal RNA gene, partial sequence (GU132497.1)	99	100	KF646012
2BD3	1	320	Streptomyces flavogriseus ATCC 33331, complete genome (CP002475.1)	98	100	KF646013
2BD11	1	386	Streptomyces violascens strain S11A-1 16S ribosomal RNA gene, partial (HQ238294.1)	99	100	KF646014
2BE8	1	453	Streptomyces sp. ME01-21h 16S ribosomal RNA gene, complete sequence (EU080953.1)	99	98	KF646015
2BF1	1	448	Streptomyces sp. MI-BOB partial 16S rRNA gene, isolate MI-BOB (FN550147.1)	99	99	KF646016
2BMA3	1	361	Nocardiopsis sp. TFS72-17 16S ribosomal RNA gene, partial sequence (HM001279.1)	99	100	KF646017
3BA6	1	455	Streptomyces parvus partial 16S rRNA gene, strain moss18, isolate (FR846235.1)	99	98	KF646018
3BA9	1	453	Streptomyces sp. 220297 16S ribosomal RNA gene, partial sequence (GU130099.1)	99	99	KF646019
3BA11	1	364	Streptomyces violascens strain S11A-1 16S ribosomal RNA gene, partial (HQ238294.1)	99	100	KF646020
3BB11	1	366	Streptomyces sp. KLBMP 1072 16S ribosomal RNA gene, partial sequence (HM153776.1)	99	100	KF646021
3BD2	1	446	Streptomyces flavogriseus partial 16S rRNA gene, strain 10, clone chit_5 (FR832623.1)	99	100	KF646022
3BD3	1	381	Streptomyces sp. TN256 partial 16S rRNA gene, strain TN256 (FN687758.1)	99	100	KF646023
3BD8	1	456	Streptomyces sp. AKB-2008-LO6 partial 16S rRNA gene, strain AKB- (AM988888.1)	99	96	KF646024
3BD10	1	448	Streptomyces drozdowiczii strain NRRL B-24297 16S ribosomal RNA gene (EF654097.1)	99	99	KF646025
3BE9	1	451	Streptomyces subrutilus strain 173270 16S ribosomal RNA gene, partial (EU570663.1)	99	98	KF646026
3BF2	1	444	Promicromonospora sp. ME02-6983B 16S ribosomal RNA gene, complete (EU080946.1)	99	100	KF646027
3BF6	1	1491	Streptomyces sp. VTT E-99-1335 (B323) 16S ribosomal RNA gene, partial sequence (AF429399.1)	99	100	KF646028
3BG8	1	376	Actinoalloteichus sp. HB272 16S ribosomal RNA gene, partial sequence (GQ863923.1)	99	99	KF646029
3BG9	1	446	Streptomyces sp. SXY49 16S ribosomal RNA gene, partial sequence (GU045532.1)	99	99	KF646030
4BA12	1	455	Streptomyces sp. AKB-2008-ET11 partial 16S rRNA gene, strain AKB (AM988883.1)	99	98	KF646031

Table S4. *Cont.*

4BB1	1	447	Streptomyces flavogriseus partial 16S rRNA gene, strain 10, clone chit_5 (FR832623.1)	99	100	KF646032
4BB9	1	450	Streptomyces albus subsp. albus gene for 16S Rrna (AB045884.1)	99	97	KF646033
4BD1	1	375	Streptomyces violascens strain G8A-24 16S ribosomal RNA gene, partial sequence (HQ238409.1)	99	100	KF646034
5BA3	1	1494	Streptomyces sp. VTT E-99-1336 (B329) 16S ribosomal RNA gene, partial sequence (AF429400.1)	99	100	KF646035
5BB4	1	447	Streptomyces sp. SXY83 16S ribosomal RNA gene, partial sequence (GU045536.1)	99	100	KF646036
5BC4	1	407	Streptomyces griseus strain ABRIINW 13 16S ribosomal RNA gene, partial (HQ597007.1)	99	100	KF646037
5BMB1	1	514	Promicromonospora sp. ME02-6983B 16S ribosomal RNA gene, complete sequence (EU080946.1)	99	87	KF646038
6BA10	1	436	Streptomyces griseus strain DSM 40931 16S ribosomal RNA gene, partial (GU383217.1)	99	99	KF646039
6BC4	1	447	Streptomyces flavogriseus partial 16S rRNA gene, strain 10, clone chit (FR832623.1)	99	100	KF646040
6BMA7	1	401	Streptomyces sp. HB110 16S ribosomal RNA gene, partial sequence (GU213497.1)	99	100	KF646041
7BA11	1	441	Streptomyces sp. HB132 16S ribosomal RNA gene, partial sequence (GQ863908.1)	98	100	KF646042
7BB12	1	448	Streptomyces sp. ME02-6979.3a 16S ribosomal RNA gene, complete (EU080943.1)	99	98	KF646043
7BC7	1	440	Streptomyces sp. L083 16S ribosomal RNA gene, partial sequence (EU135902.1)	99	99	KF646044
7BD3	1	382	Streptomyces sp. MS218 16S ribosomal RNA gene, partial sequence (EU908199.1)	98	100	KF646045
7BD4	1	509	Streptomyces luridiscabiei strain S63 16S ribosomal RNA, complete sequence (NR_025155.1)	100	87	KF646000
8BA1	1	459	Streptomyces albidoflavus strain HD-109 16S ribosomal RNA gene, partial (EF620361.1)	99	99	KF646046
8BC4	1	458	Streptomyces sp. MS1/7 16S ribosomal RNA gene, partial sequence (AY550275.2)	98	99	KF646047
8BC6	1	452	Streptomyces drozdowiczii partial 16S Rrna gene, isolate PhyCEm-1258 (AM921645.1)	99	99	KF646048

Table S5. Composition of sequence groups consisting of strains which share >99% 16S rRNA sequence identity.

Sequence group	Number of strains	Strains in sequence group<br>*Isolate location denoted by first two characters of strain name*
1	123	1BA1, 1BA4, 1BA5, 1BA6, 1BA7, 1BA9, 1BA11, 1BB4, 1BB5, 1BB6, 1BB10, 1BB11, 1BC11, 1BC2, 1BC3, 1BC5, 1BD1, 1BD7, 1BD10, 1BE7, 1BF1, 2BA4, 2BA5, 2BA7, 2BA10, 2BB4, 2BB6, 2BB12, 2BC2, 2BC3, 2BC7, 2BC8, 2BC12, 2BD4, 2BD9, 2BD10, 2BE2, 2BE3, 2BE4, 2BE5, 2BE7, 2BE9, 2BF3, 2BF5, 2BF8, 2BF9, 2BF12, 2BG1, 3BA1, 3BA4, 3BA12, 3BB7, 3BB8, 3BC3, 3BC4, 3BD1, 3BE2, 3BE3, 3BE4, 3BE5, 3BE11, 3BF1, 3BF3, 3BF5, 3BG3, 3BG6, 3BF10, 4BA5, 4BA9, 4BB2, 4BB12, 4BC4, 4BC5, 4BC7, 4BC8, 4BC10, 4BD3, 4BD4, 4BD9, 5BA8, 5BA10, 5BB7, 5BC5, 5BC7, 5BC9, 5BD2, 5BD5, 5BD6, 5BD7, 6BA6, 6BB2, 6BC1, 6BC2, 6BC10, 6BC11, 6BMA8, 7BB1, 7BC5, 7BC9, 7BC12, 7BD5, 7BD6, 7BD8, 7BD11, 7BD12, 7BE3, 7BE4, 7BE11, 7BF4, 7BF6, 7BF10, 7BG3, 7BG4, 7BG10, 7BMA9, 8BA3, 8BA4, 8BA5, 8BA11, 8BB11, 8BB12, 8BC2, 8BC3
2	2	1BA12, 1BD8
3	5	1BA2, 1BA8, 3BD4, 7BF3, 8BB5
4	19	1BB12, 1BE10, 2BA1, 2BA8, 3BA5, 3BC7, 3BG10, 4BA7, 4BA11, 4BD10, 4BD8, 5BA2, 5BB8, 5BB11, 6BA1, 6BC7, 7BG6, 8BB1, 8BB4
5	4	1BB7, 1BB9, 7BC8, 7BG8
6	53	1BA10, 1BB8, 1BC4, 1BC6, 1BC9, 1BD12, 1BE2, 1BE4, 2BA9, 2BA11, 2BB8, 2BC9, 2BD8, 2BD12, 2BE1, 2BE10, 2BE12, 2BF10, 3BA3, 3BA7, 3BA8, 3BB5, 3BB9, 3BB10, 3BC6, 3BC12, 3BD5, 4BA6, 4BC11, 4BD6, 5BA4, 5BC6, 5BC10, 5BMA6, 6BA2, 6BA4, 6BA8, 6BB5, 6BC9, 7BA4, 7BA6, 7BA7, 7BB2, 7BB6, 7BD2, 7BF1, 7BF8, 7BF9, 7BG5, 7BG7, 8BA7, 8BA8, 8BC7
7	7	1BD4, 4BB10, 4BE1, 5BA12, 7BA8, 7BA9, 7BF5
8	13	1BB3, 1BD5, 3BB4, 3BC11, 3BE10, 4BB7, 4BC1, 4BE2, 5BB3, 5BC11, 5BD8, 6BB1, 6BC3
9	20	1BC8, 1BE6, 1BE8, 1BE12, 2BA6, 2BC1, 2BD1, 2BF7<br>3BG4, 4BB11, 5BA9, 5BB9, 6BB4, 6BC8, 7BA3, 7BB8, 7BC11, 7BD7, 7BE10, 7BG2
10	7	2BA3, 2BB2, 3BB2, 3BD12, 3BF12, 6BA11, 6BC12
11	2	2BB7, 3BG7
12	7	3BA10, 3BB3, 3BE1, 3BF9, 3BG5, 4BC6, 5BB2
13	11	2BC5, 2BD6, 2BF4, 3BA2, 3BB1, 3BB6, 3BB12, 4BA10, 4BD12, 5BC12, 7BF12
14	2	3BC5, 3BG2
15	5	3BC8, 4BD11, 5BA5, 6BB3, 6BB11
16	5	2BG3, 3BC9, 3BD6, 3BE12, 6BA5
17	4	3BD9, 3BE8, 5BB5, 5BC2
18	3	2BC4, 3BF8, 6BC5

Table S5. *Cont.*

19	12	2BB10, 3BD7, 3BE7, 3BF7, 4BB5, 4BB6, 4BC12, 5BA7, 5BB12, 5BD1, 6BA9, 6BC6
20	5	4BC2, 6BA3, 6BA12, 6BB8, 6BB9
21	2	4BB8, 4BD5
22	2	1BMA2, 4BD7
23	6	2BB1, 2BE6, 3BF4, 4BA3, 5BA1, 5BC8
24	3	4BD2, 5BD4, 7BE9
25	2	5BA11, 6BA7
26	11	2BB3, 2BC6, 2BF6, 5BD3, 6BB12, 7BB7, 7BD10, 7BE2, 7BE7, 7BE12, 7BG1
27	2	5BC1, 6BB6
28	3	6BB7, 7BA1, 7BA12
29	14	2BA2, 2BB9, 3BE6, 4BA1, 4BA8, 5BA6, 5BC3, 6BB10, 7BA10, 7BB5, 7BB11, 7BC4, 7BC6, 7BC10
30	2	7BD9, 7BE6
31	6	1BD3, 8BA9, 8BB6, 8BB8, 8BC9, 8BC10
32	4	5BB1, 7BE8, 7BF11, 8BB10
33	6	3BC10, 7BC1, 7BE5, 7BD1, 8BA6, 8BB2
34	7	1BD9, 2BC10, 2BG2, 5BB6, 7BC2, 7BE1, 8BB3
35	9	1BB2, 3BC1, 3BC2, 3BD11, 8BA10, 8BB7, 8BB9, 8BC1, 8BC5
36	20	1BC12, 1BE9, 1BMA1, 2BB5, 2BD5, 2BF2, 2BE11, 3BG1, 3BMA4, 4BA4, 4BC9, 5BB10, 5BMB2, 7BA2, 7BA5, 7BB3, 7BB9, 7BG9, 8BA12, 8BC8
37	4	3BF11, 4BC3, 7BB4, 7BC3
38	3	4BB3, 4BB4, 7BB10
39	2	2BF11, 4BA2

Figure S1. Canonical correspondence analysis (CCA) of the relationship between culture-independent species level community composition (in black: OTUs identified using mothur) and sediment parameters (in red: % Organic Matter (OM), pH, P_2O_2, K_2O, Ca and Mg) by site (in green: Black Beach (1B, 2B, 3B), Chance Harbour (4B, 5B, 6B), Pocologan (7B) and Mispec Bay (8B)).

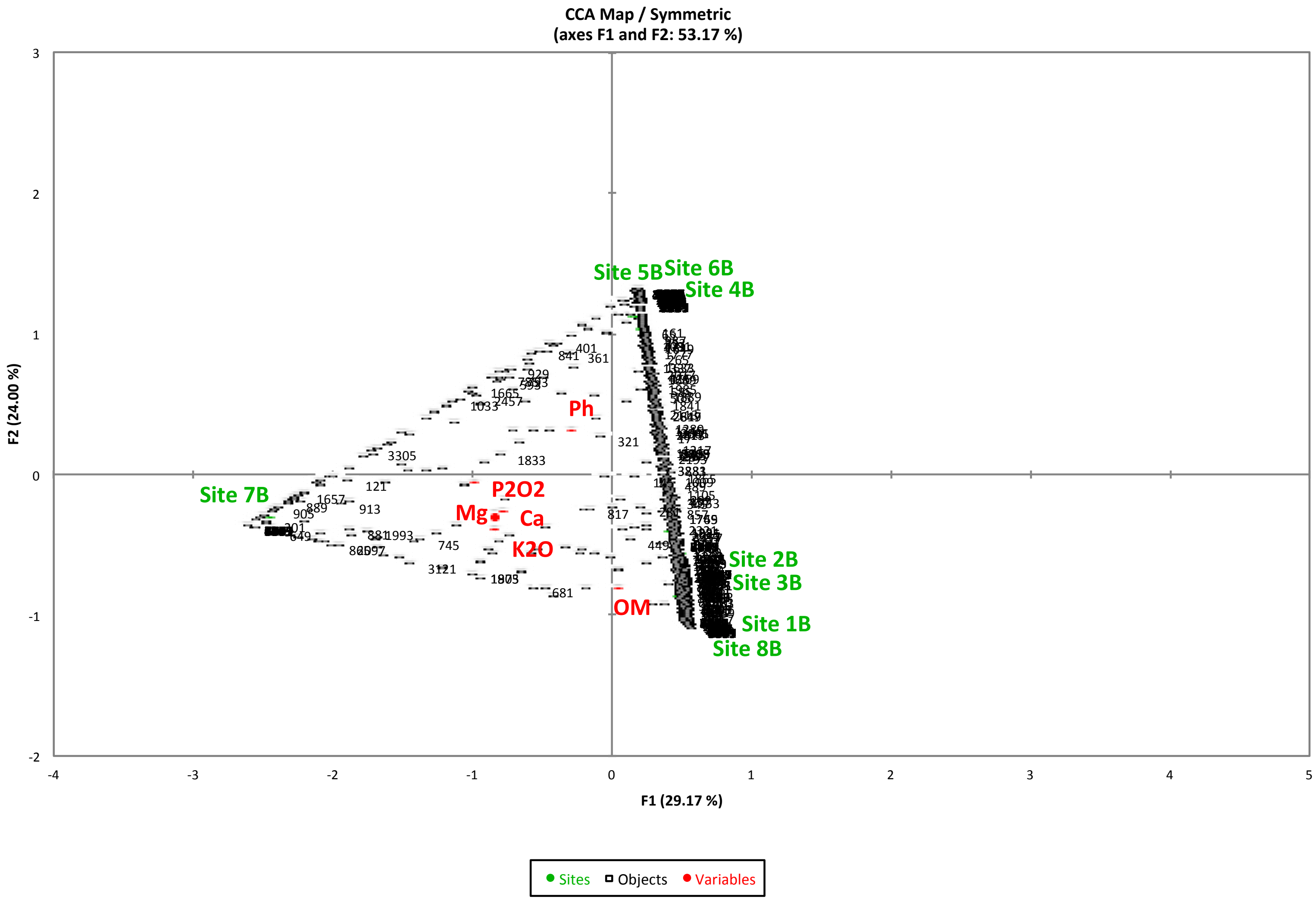

Figure S2. Canonical correspondence analysis (CCA) of the relationship between culture-independent species level community composition (in black: OTUs identified using mothur) and the sediment parameters (in red: B, Cu, Fe, Na, Zn, S) by site (in green: Black Beach (1B, 2B, 3B), Chance Harbour (4B, 5B, 6B), Pocologan (7B) and Mispec Bay (8B)).

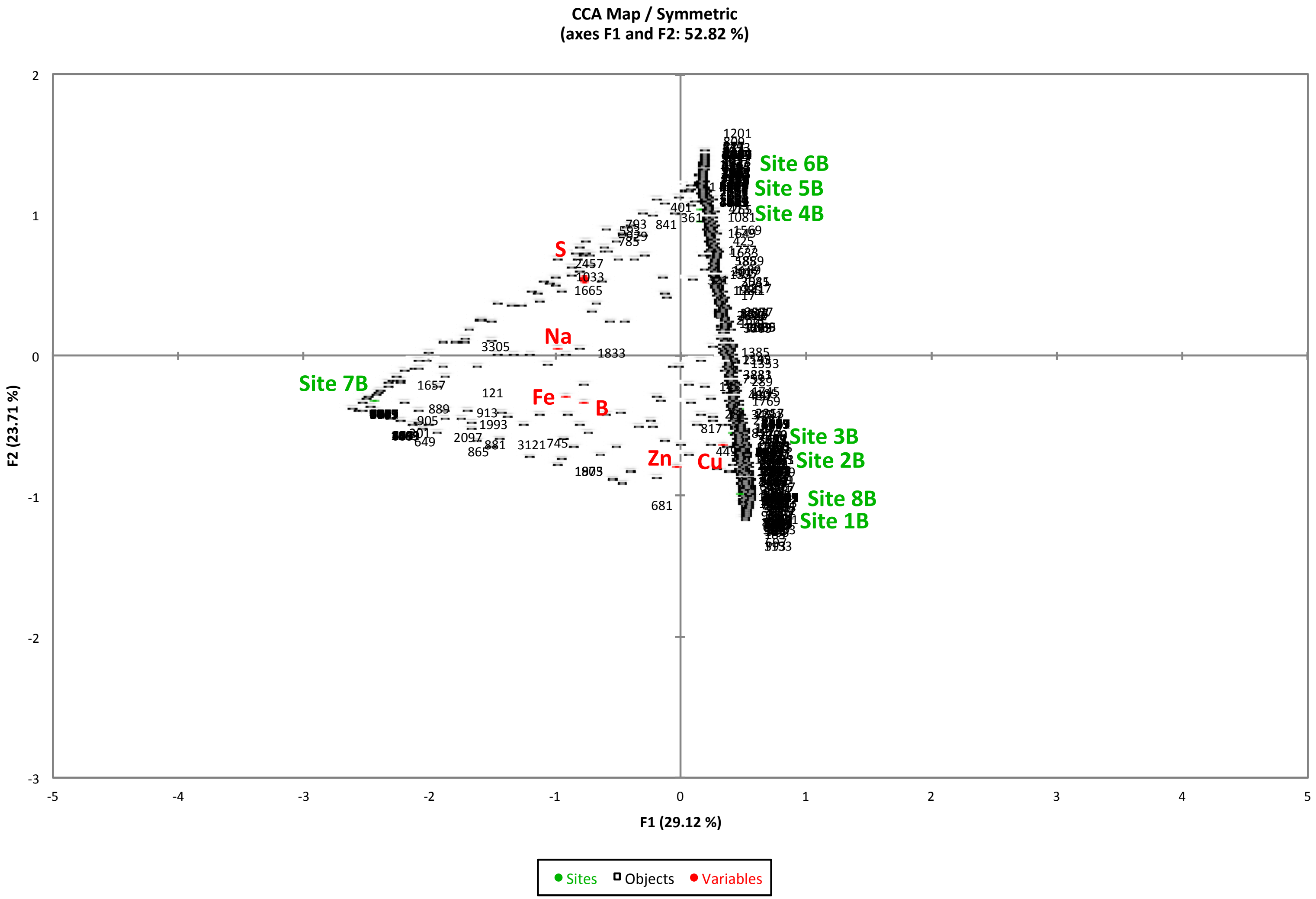

Figure S3. Canonical correspondence analysis (CCA) of the relationship between culture-independent species level community composition (in black: OTUs identified using mothur) and the sediment parameters (in red: sediment particle size: size 1 (<0.6 mm), size 2 (0.6–1 mm), size 3 (1–2 mm) and size 4 (>2 mm)) by site (in green: Black Beach (1B, 2B, 3B), Chance Harbour (4B, 5B, 6B), Pocologan (7B) and Mispec Bay (8B)).

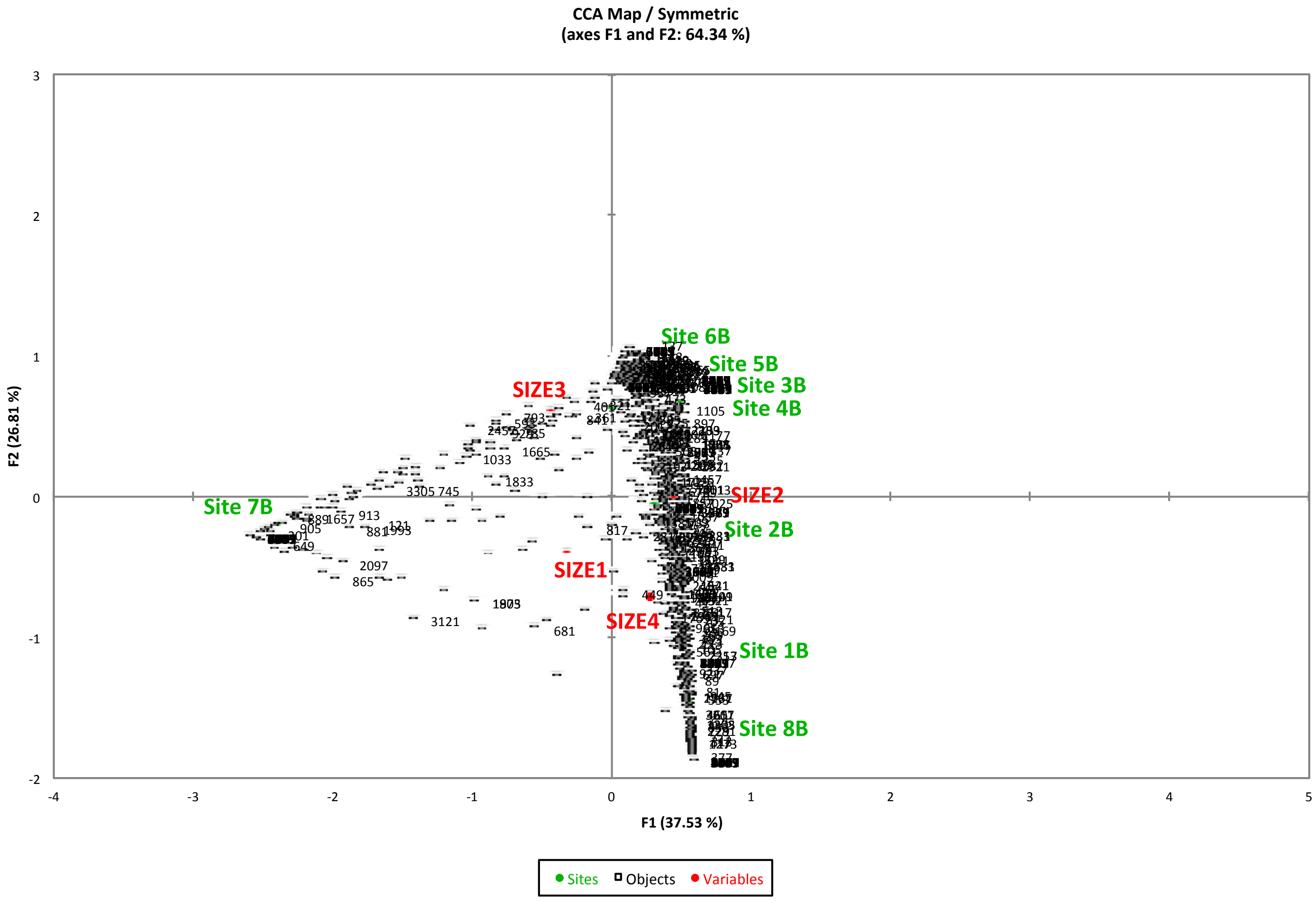

Figure S4. Canonical correspondence analysis (CCA) of the relationship between culture-dependent species level community composition (in black: OTUs based on 99% sequence identity) and the sediment parameters (in red: % Organic Matter (OM), pH, P_2O_2, K_2O, Ca and Mg) by site (in green: Black Beach (1B, 2B, 3B), Chance Harbour (4B, 5B, 6B), Pocologan (7B) and Mispec Bay (8B)).

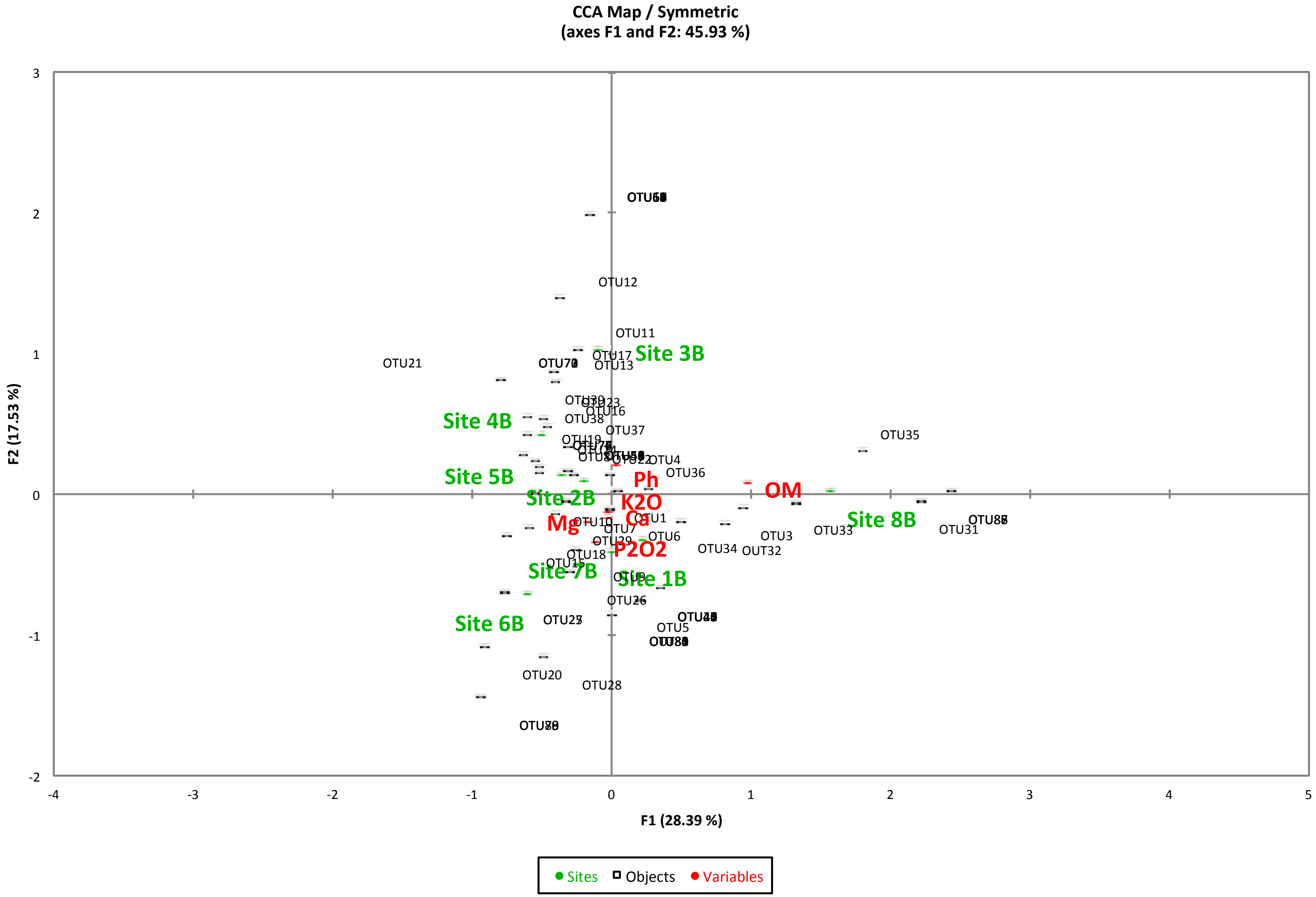

Figure S5. Canonical correspondence analysis (CCA) of the relationship between culture-dependent species level community composition (in black: OTUs based on 99% sequence identity) and the sediment parameters (in red: B, Cu, Fe, Na, Zn, S) by site (in green: Black Beach (1B, 2B, 3B), Chance Harbour (4B, 5B, 6B), Pocologan (7B) and Mispec Bay (8B)).

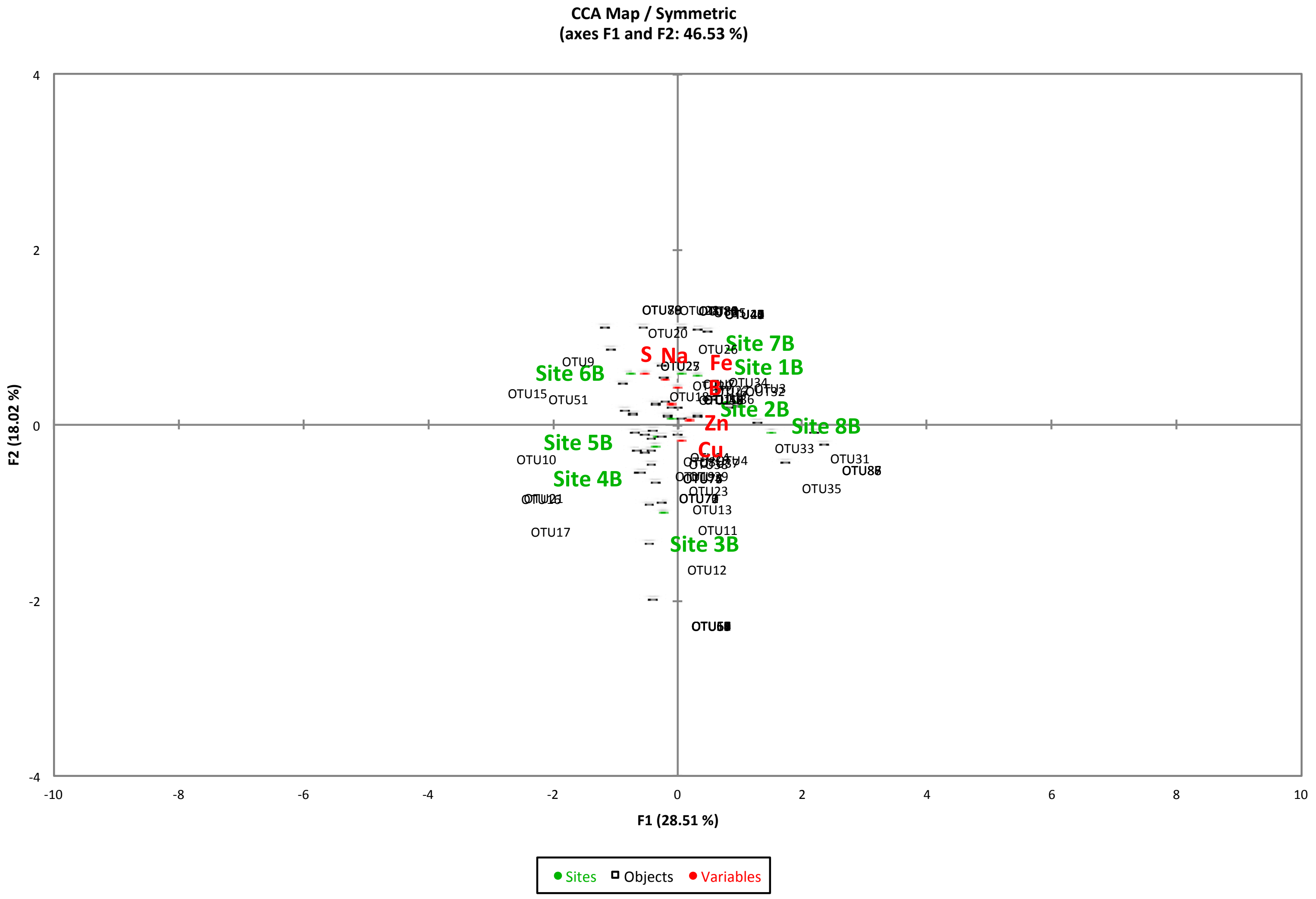

Figure S6. Canonical correspondence analysis (CCA) of the relationship between culture-dependent species level community composition (in black: OTUs based on 99% sequence identity) and the sediment parameters (in red: sediment particle size: size 1 (<0.6 mm), size 2 (0.6–1 mm), size 3 (1–2 mm) and size 4 (>2 mm)) by site (in green: Black Beach (1B, 2B, 3B), Chance Harbour (4B, 5B, 6B), Pocologan (7B) and Mispec Bay (8B)).

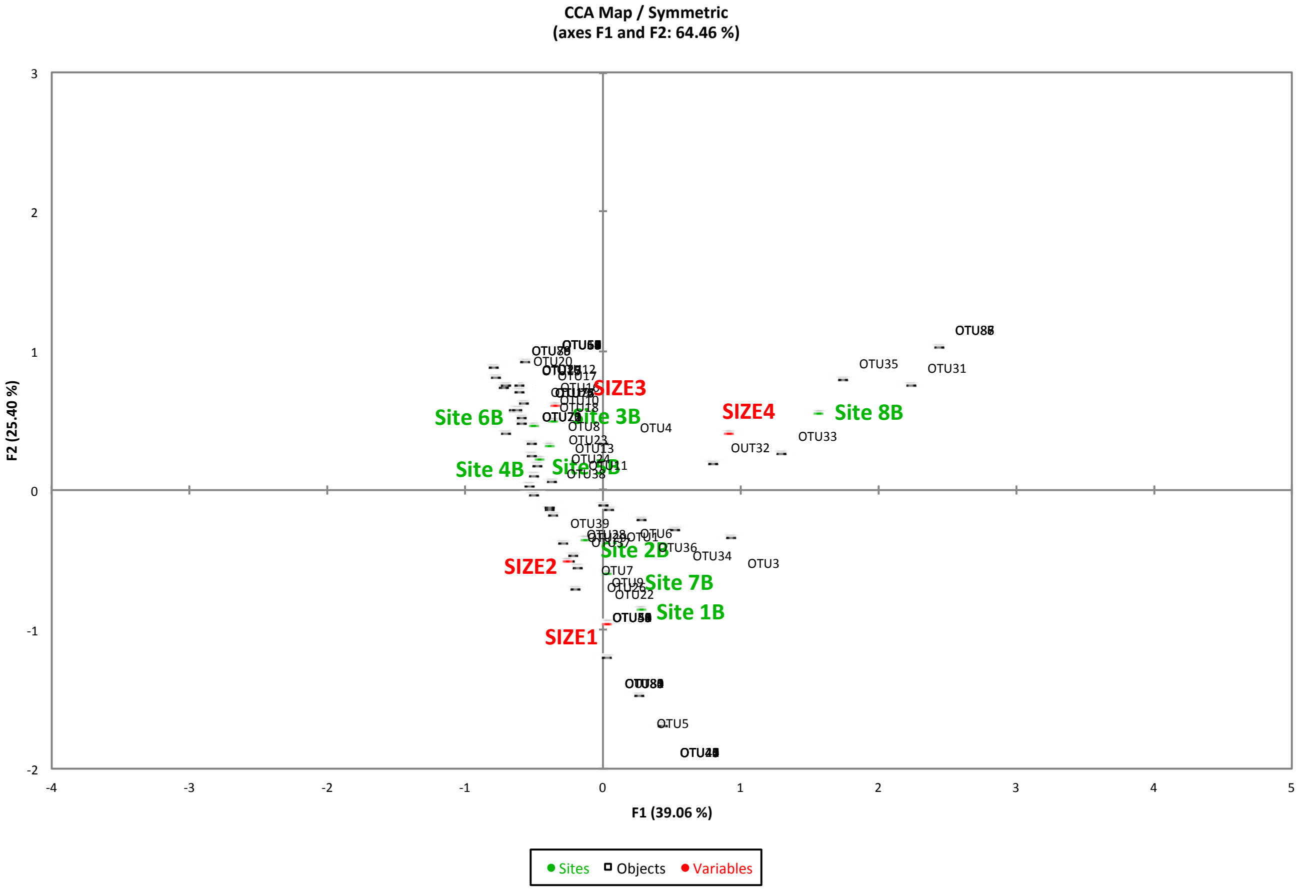

© 2014 by the authors; licensee MDPI, Basel, Switzerland. This article is an open access article distributed under the terms and conditions of the Creative Commons Attribution license (http://creativecommons.org/licenses/by/3.0/).
